# Experiences and needs of front-line nurses during the COVID-19 pandemic: A systematic review and qualitative meta-synthesis

**DOI:** 10.3389/fpubh.2022.805631

**Published:** 2022-07-22

**Authors:** Shenglan Ding, Shuhua Deng, Yilan Zhang, Qingxia Wang, Zhiping Liu, Jing Huang, Xiaorong Yang

**Affiliations:** ^1^Department of Nursing, Chengdu Women's and Children's Central Hospital, School of Medicine, University of Electronic Science and Technology of China, Chengdu, China; ^2^Department of Respiratory, Chengdu Integrated Traditional Chinese Medicine (TCM) and Western Medicine Hospital, Chengdu, China; ^3^Delivery Room, Chengdu Women's and Children's Central Hospital, School of Medicine, University of Electronic Science and Technology of China, Chengdu, China; ^4^Department of Endocrinology, The First Affiliated Hospital of Chongqing Medical University, Chongqing, China; ^5^Department of Rehabilitation, Chengdu Women's and Children's Central Hospital, School of Medicine, University of Electronic Science and Technology of China, Chengdu, China

**Keywords:** nurses, coronavirus disease 2019, experiences, needs, qualitative meta-synthesis, systematic review

## Abstract

**Background:**

Front-line nurses have played a critical role during the coronavirus disease 2019 (COVID-19) pandemic. A number of qualitative studies reported front-line nurses' experiences and needs in caring for patients with COVID-19. However, the application of evidence from a single qualitative study to guide clinical practice has limitations. This study aimed to explore front-line nurses' experiences and needs during the COVID-19 pandemic through a qualitative meta-synthesis.

**Methods:**

Seven databases were searched from 1 December 2019 to 20 January 2022, including PubMed, Web of Science, Cochrane COVID-19 study register, CINAHL, PsycINFO, MedRxiv, and bioRxiv. The quality of included studies was appraised using the Critical Appraisal Skills Program (CASP) qualitative research appraisal tool. Meta-synthesis was used to synthesize the data from included studies.

**Results:**

A total of 70 studies were included, and five synthesized findings were developed: (1) Although nurses actively devoted themselves to fighting against COVID-19, considering their professional responsibility and historical previous experience with mankind, they were not invulnerable; (2) There were various difficulties and challenges in caring for patients with COVID-19, including fear related to providing patients with care, shortage of protective equipment and manpower, and negative attitude of family members; (3) Facing difficulties and challenges, nurses could only partly cope by using mixed means to overcome those, including media, learning, gaining skills, responding together, and organizational assistance; (4) To better respond to the COVID-19 pandemic, nurses' needs should be paid attention to. Counseling, training, information, resources, and investment are pivotal; (5) Despite the hardships, nurses became stronger and gained gratitude, positivity, mental peace, and confidence.

**Conclusions:**

This study reveals that the psychological experiences of front-line nurses varied, and they faced a variety of challenges. Although nurses had some coping strategies, they still needed multifaceted support to meet the challenges.

**Systematic review registration:**

https://www.crd.york.ac.uk/PROSPERO/, PROSPERO: CRD42021255468.

## Introduction

In December 2019, a novel pneumonia with unknown clinical and therapeutic aspects emerged in Wuhan, Hubei Province (1). Subsequently, this infectious disease was named as coronavirus disease 2019 (COVID-19) by the World Health Organization (WHO) (2). The COVID-19 caused by severe acute respiratory syndrome coronavirus 2 (SARS-CoV-2) is spreading around the world rapidly and poses a significant threat to global health (3). Up to March 6, 2022, there had been 443,895,905 confirmed patients with COVID-19 and 5,993,901 deaths (4). As of this date, the number of infected and suspected cases and mortality is still increasing.

Early in the pandemic, Hubei, China was the worst infection area in the world, and the government assigned more than 42,000 medical staff to assist Hubei in responding to the sudden crisis. Notably, nurses accounted for 68% of the total number of medical staff (5). As the backbone during the COVID-19 pandemic, front-line nurses around the world have played a critical role in the nursing care and management of patients with COVID-19. The front-line nurses caring for patients with COVID-19 had a moderate to high level of fatigue, poor quality of care, higher intention to quit their job, and lower work satisfaction (6, 7), which might affect the nurses' mental health and patient safety outcomes in turn. The front-line nurses provide direct care for the patients with COVID-19, which poses a high risk of being infected for them. A great number of medical staff have been infected or died as a result of COVID-19. WHO estimated that between 80,000 and 180,000 medical workers, including nurses, died from COVID-19 between January 2020 and May 2021 (8).

To obtain a rich detail of front-line nurses' experiences, some researchers carried out qualitative studies. For some nurses, front-line work had negative impacts on their mental status during the COVID-19 pandemic (9–11). Meanwhile, the nurses faced many challenges, such as lacking personal protective equipment (PPE) and working experience in infectious disease (12–14). However, the application of evidence collected from a single qualitative study to guide clinical practice has limitations.

To better understand front-line nurses' experiences for further practice, qualitative meta-synthesis needs to be performed. Recently, Joo and Liu (15) conducted a qualitative systematic review in August 2020 to synthesize the qualitative data of nurses' experiences during the COVID-19 pandemic. In this systematic review, six of nine included studies were from China, and qualitative studies from other countries in this field were limited. During the COVID-19 pandemic, knowledge and healthcare issues are regularly changing with the progression of time due to the highly evolving nature of the virus and fluctuation of the number of patients. New qualitative studies exploring the experiences of front-line nurses have been published and involve new evidence (12, 14, 16). Therefore, we performed this meta-synthesis of qualitative studies to gain a deeper understanding of front-line nurses' experiences during the COVID-19 pandemic. More available evidence from the different populations and regions will be included, and the sample size will be expanded. This will not only further enrich the review of Joo et al. but also better guide future clinical practice and nursing research in the similar crises.

## Methods

This meta-synthesis has been conducted according to the reporting guideline, Enhancing Transparency in Reporting the Synthesis of Qualitative Research (ENTREQ) (17). The protocol for this review was evaluated by all the authors. The protocol is registered in PROSPERO (reference No. CRD42021255468). The research question was guided by the SPIDER tool (Sample, Phenomenon of Interest, Design, Evaluation, Research Type), which was developed from PICO (Population, Intervention, Comparison, Outcome) (18). The main research questions were: (1) what are the experiences and perceptions of front-line nurses during the outbreak of COVID-19? and (2) What kind of support do front-line nurses need to respond to the COVID-19 pandemic?

### Search strategy

For this review, a systematic literature search was conducted by two authors (SD1 and SD2) to ascertain studies about experiences of front-line nurses caring for patients with COVID-19 on PubMed, Web of Science, Cochrane COVID-19 study register, CINAHL, and PsycINFO. MedRxiv and bioRxiv were also searched for gray literature. The SPIDER tool was used to frame the search terms (18) (Table 1). The search terms included: nurs^*^; 2019 novel coronavirus disease, COVID-19, SARS CoV-2 infection, COVID-19 virus disease, 2019 novel coronavirus infection, 2019-nCoV infection, coronavirus disease-19, COVID-19 pandemic, COVID-19 virus infection, 2019-nCoV disease; interview^*^, focus group^*^, view^*^, experience^*^, perception^*^, need^*^, feel^*^; qualitative research and mixed methods. Truncation symbols and Boolean operators (“or” and “and”) were used to combine synonyms and index terms. The search terms were modified in the databases, and search results were refined using filters. All databases were searched from 1 December 2019 to 20 January 2022. The reference lists of included studies were screened manually to identify additional possible studies not identified in the electronic search.

**Table 1:** Search terms used in systematic literature search.

**Domain**	**Search terms**
S = Sample	Nurs*
PI = Phenomenonof interest	*COVID-19* OR *COVID19*OR*2019 novel coronavirus disease* OR*SARS-CoV-2 infection* OR*COVID-19 virus disease* OR *2019 novel coronavirus infection* OR*2019-nCoV infection* OR *coronavirus disease 2019* OR*coronavirus disease-19* OR 2019-nCoV disease OR *COVID-19 virus infection*OR *2019 novel coronavirus pneumonia* OR *2019 Novel Coronavirus–Infected Pneumonia*
D = Design	Interview*OR focus group*
E = Evaluation	View*OR experience*OR perception*OR need*ORfeel*
R = Researchtype	*Qualitative research OR mixed methods*

### Inclusion and exclusion criteria

Studies were included if they met the following criteria: (a) explore experiences of front-line nurses caring for patients with COVID-19; (b) primary qualitative studies (including the qualitative component of mixed-methods studies); (c) published in English between 2019 and 2022; (d) peer-reviewed journal articles or preprints. Exclusion criteria were as follows: (a) review articles, editorials, clinical case reports, or commentary articles; (b) no full-text or republished articles; (c) quantitative studies or mixed studies focusing on quantitative studies.

### Screening

All articles were assessed by two authors (SD1 and SD2) independently according to the inclusion and exclusion criteria. Any disagreements regarding studies selection were resolved by discussion or by resorting to the judgment of a third author (JH) when needed. If the eligibility was still not clear, the entire research team discussed to reach a consensus.

### Quality assessment

The quality of included studies was evaluated by the Critical Appraisal Skills Program (CASP) with ten criteria (19). The studies were assessed for the statements of study aims, appropriate qualitative methodology, research designs, recruitment strategies, data collections, reflexivity of the researchers, ethical considerations, rigors of data analyses, statements of the findings, and values of the research. The quality assessment was conducted by two authors (SD1 and SD2) independently, and, in cases where there was no agreement, the third author (JH) mediated.

### Data extraction

Two authors (SD1 and SD2) collected the following information: first author, year of publication, country, study aim, study design, time period of study, sampling, sample characteristics, data collection and analysis, and key findings. A standardized form was used to extract data of the included studies. Any discrepancies were resolved by discussion between the authors or by resorting to the judgment of a third author (JH) until a consensus was reached.

### Data synthesis

Meta-aggregation was employed in this study to synthesize the findings of included studies. This synthesis approach was done in a three-stage process: (1) the authors repeatedly read the included studies and extracted all the relevant findings; (2) the authors repeatedly read and analyzed the findings from included studies and categorized them on the basis of similarity in meaning; (3) the authors grouped categories into synthesized themes (20). For qualitative data, there are three levels of credibility: (1) Unequivocal (U)—relates to evidence beyond a reasonable doubt, which may include findings that are matter of fact, directly reported/observed and not open to challenge; (2) Credible (C)—those that are, albeit interpretations, plausible in light of data and the theoretical framework. They can be logically inferred from the data; (3) Not Supported (NS) – when 1 nor 2 apply and when most notable findings are not supported by the data (21). The System for the Unified Management, Assessment and Review of Information (SUMARI) of the Joanna Briggs Institute (JBI) has been used to assist authors in this process.

## Results

### Literature selection

**Figure 1:**
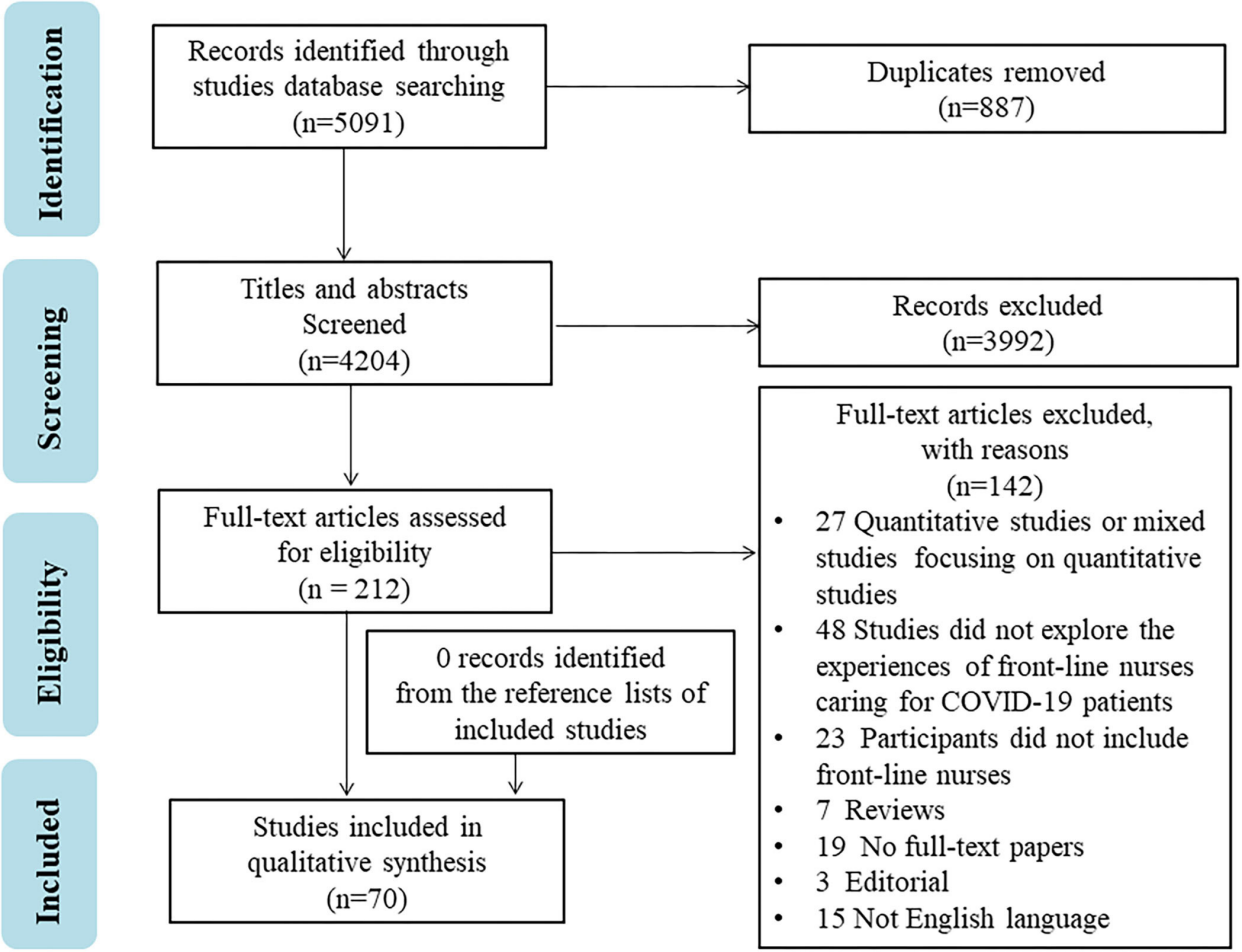
Flow chart of search results and study selection.

A total of 5,091 studies were identified. After removing duplicates by importing into Note-Express software, 887 duplicate publications were identified and removed. The remaining 4,204 studies were screened by reading titles and abstracts, and 212 studies met the inclusion criteria. After screening the full text, 70 studies were finally included (Figure 1).

### Characteristics of the studies

Of the 70 included studies, nineteen were from China (13, 22–39), sixteen were from Iran (9, 11, 12, 40–52), eight were from Turkey (53–60), seven were from US (61–67), three were from Italy (10, 14, 68), two were from Spain (69, 70), two were from South Korea (16, 71), two were from Canada (72, 73), two were from Brazil (74, 75), two were from the Philippines (76, 77), and the remaining seven were from Japan, Thailand, Indonesia, Demark, Lebanon, Qatar, and Sri Lanka, respectively (78–84). Sample sizes varied from 9 to 719 participants, and the participants of 2 included studies contained a small number of physicians (12, 22). The most common study design was a qualitative study using semi-structured interviews for data collection. Most studies were conducted between January and October 2020, which was in the first wave of the pandemic. Detailed characteristics of included studies are described in Table 2.

**Table 2:** Detailed characteristics of included studies.

**References, country**	**Aim of study**	**Study design and sampling**	**Time period of study**	**Sample characteristics**	**Data collection and analysis**	**Key findings**
Sun et al. (26), China	To explore the psychology of nurses caring for COVID-19 patients	Qualitative study; purposeful sampling	First wave of pandemic (from January 20 to February 10, 2020)	Nurses (*n* = 20;age group: 25–49; working unit: negative pressure ward; working experience: 5.85 ±6.43 years)	One-to-one interviews; Colaizzi's phenomenological analysis	1. Significant amount of negative emotions in the early stage 2. Coping and self-care styles 3. Growth under pressure 4. Positive emotions occurred simultaneously or progressively with negative emotions
Liu et al. (22), China	To describe the experiences of health-care providers in the early stages of the COVID-19 outbreak	Qualitative study; purposive and snowball sampling	First wave of pandemic(from February 10 to February 15,2020)	Nurses and physicians (*n* = 9,4; age group: 22–42; working unit: COVID-19 ward; working experience: 2–17 years)	Semi-structured, in-depth telephone interviews; Haase's adaptation of Colaizzi's method	1. “Being fully responsible for patients' wellbeing—‘this is my duty”' 2. Challenges of working on COVID-19 wards 3. Resilience amid challenges
Zhang et al. (27), China	To identify the psychological change process of the registered nurses in theCOVID-19 outbreak	Qualitative descriptive study; purposive sampling	First wave of pandemic (February 9 to March 15, 2020)	Nurses (*n* = 23;average age of31.5; working unit: COVID-19 isolation ward; working experience: 2–20 years)	Semi-structured interviews; Colaizzi's method	1. Early stage—being ambivalent 2. Middle stage—emotional exhaustion 3. Later stage—energy renewal
Fan et al. (24), China	To investigate the experiences, vocational issues and psychological stresses of front-line nurses fighting against the COVID-19 outbreak	Qualitative descriptive study; purposeful sampling	First wave of pandemic	Transdisciplinary nurses and non-transdisciplinary nurses (*n* = 25, *n* = 19; age group over 20; working unit:COVID-19 ward; working experience: 1 to more than 15 years)	Semi-structured face-to-face interviews; the Braun Clarke Thematic Analysis	1. Awareness of nurses' responsibilities and roles 2. Recognition of responsibilities of transdisciplinary nursing work 3. Psychological problems caused by transdisciplinary work
Liu et al. (25), China	To explore the experiences of front-line nurses combatingCOVID-19 outbreak	Qualitative study; maximum difference sampling	First wave of pandemic(January 26 to February 5, 2020)	Nurses (*n* = 15,average age of 27.83 ± 5.4; working unit: general ward, infectious disease ward, ICU; working experience: 7.30 ± 5.62 years)	Structured in-depth interviews; standard qualitative methods	1. Facing tremendous challenges and danger 2. Strong pressure because of COVID-19 3. Strong responsibility and identity as a health care provider 4. Rational understanding of the epidemic
Kackin et al. (53), Turkey	To explore the experiences and psychosocial problems of nurses caring forCOVID-19 patients	Qualitative study; purposive sampling	First wave of pandemic(May9 to May12, 2020)	Nurses (*n* = 10,age group 24–40;) working unit: COVID-19 ward; working experience: NA)	Semi-structured in-depth interviews; Colaizzi's phenomenological analysis	1. Effects of the outbreak; 2. Short-term coping strategies 3. Needs
Catania et al. (14), Italy	To explore nursing management issues within COVID-19 narratives of Italian front-line nurses	Qualitative study; NA	First wave of pandemic(April15 to May16, 2020)	Nurses (*n* = 23,age group 22–59; working unit: emergency department, infectious disease department, sub-ICU, ICU; working experience: 18.04 ± 13.83 years)	A web link disseminated *via* institutional websites and some open questions to guide the participants' narrations; thematic analysis	1. Organizational and logistic change 2. Leadership models adopted to manage the emergency 3. changes in nursing approaches 4. Personal protective equipment (PPE) issues 5. Physical and psychological impact on nurses 6. Team value/spirit
Sadati et al. (11), Iran	To investigate nurses' perceptions and experiences of COVID-19 outbreak	Qualitative study; purposive sampling	First wave of pandemic(March 2020)	Nurses (*n* = 24; age group: NA; working unit: COVID-19 ward; working experience: NA)	Semi-structured interviews; inductive and deductive thematic analysis	1. Defected preparedness 2. The worst perceived risk 3. Family protection 4. Social stigma 5. Sacrificial commitment
Alizadeh et al. (12), Iran	To explore psychological distress experienced by Iranian health-care providers in the COVID-19 outbreak	Qualitative study; purposive sampling	First wave of pandemic(February 29 to March 20, 2020)	Physicians and nurses (*n* = 6,12;age group:24–42; working unit: COVID-19 ward; working experience: NA)	Semi-structured interviews; content analysis method	1. Occupational demands 2. Supportive resources
He et al. (13), China	To examine the experiences of Chinese nurses during the very first period of COVID-19 outbreak	Qualitative study; convenience sampling and snowball	First wave of pandemic(February 2020)	Nurses (*n* = 10;age group 22–43; working unit: COVID-19 ward; working experience: 2–23 years)	In-depth and semi structured interviews; content analysis	1. Different psychological stages experienced 2. Work stress and new challenges 3. New concepts of caring for patients
Galehdar et al. (40), Iran	To explore nurses' experiences of psychological distress during COVID-19 outbreak	Qualitative study; purposeful sampling	First wave of pandemic(March to May, 2020)	Nurses (*n* = 20;average age of 31.95 ± 6.64; working unit: COVID-19 ward; working experience: 7.25 ± 5.9 years)	Semi-structured in depth telephone interviews; Graneheim and Lundman's content analysis	1. Death anxiety 2. Anxiety due to the nature of the disease 3. Anxiety related to corpse burial 4. Fear of infecting the family5. Distress about time wasting 6. Emotional distress for delivering bad news 7. Fear of being contaminated 8. The emergence of obsessive thoughts 9. The bad feeling of wearing personal protective equipment 10. Conflict between fear and conscience 11. Public ignorance of preventive measures
Galehdar et al. (41), Iran	To explore nurses' perception of taking care of patients with COVID-19	Qualitative study; purposeful sampling	First wave of pandemic(March to April, 2020)	Nurses (*n* = 13;average age of 33± 11.4; working unit: COVID-19 ward; working experience: 13 ± 8.69 years)	Semi-structured in-depth telephone interviews; content analysis	1. Care erosion 2. Nursing professional growth 3. Necessities
Karimi et al. (9), Iran	To explore the lived experiences of nurses caring for patients with COVID-19	Qualitative study; purposeful sampling	First wave of pandemic	Nurses (*n* = 12;average age of 29.41 ± 2.72; working unit: COVID-19 ward; working experience: 6.75 ± 2.52 years)	Semi-structured interview; descriptive Colaizzi method	1. Mental condition 2. Emotional condition 3. Care context
Ohta et al. (78), Japan	To explore nurses' changing perceptions with regard to the efforts in preparation for working in a COVID-19 ward	Qualitative study; NA	First wave of pandemic	Nurses (*n* = 16;age group: NA; working unit: COVID-19 ward; working experience: NA)	Ethnography and semi-structured interviews; Grounded theory	1. Pre–COVID-work perceptions and fear, 2. Overcoming fear 3. Shadow cast by working in the COVID-19 ward 4. An integrated approach to the fear of COVID-19
Yin et al. (29), China	To explore the psychological needs of nurses caring for patients with COVID-19	Qualitative study; purposive sampling	First wave of pandemic	Nurses (*n* = 10;average age of 29.9 ± 3.6; working unit: COVID-19 ward; working experience: <5 years: *n* = 3, ≥5 years: *n* = 7)	Semi-structured, personal, in-depth interview; category analysis	1. The existence needs 2. The relatedness needs 3. The growth needs
Arcadi et al. (68), Italy	To explore the experience of Italian nurses engaged in caring for patients with COVID-19	Qualitative study; Purposive sampling	First wave of pandemic(March to April 2020)	Nurses (*n* = 20;average age of 26.25 ± 3.30; working unit: emergency department; working experience: ≤ 6 months in emergency departments)	Semi-structured face-to-face interviews; Hermeneutic Phenomenological analysis process	1. Uncertainty and fear 2. Alteration of perceptions of time and space 3. Change in the meaning of “to care” 4. Changes in roles and relationships
Schroeder et al. (61), US	To explore the experience of being a registered nurse caring for patients with COVID-19 during the early stages of the pandemic	Qualitative study; purposive convenience sampling	First wave of pandemic(March and April, 2020)	Nurses (*n* = 21;average age of 33.5 ± 7.3; working unit: emergency room, critical care, medical-surgical units and float pool; working experience: 7.9 ± 6.6 years)	One-time semi-structured in-person interviews; content analysis	1. Structures: adjusting to a dynamic COVID-19 context 2. Processes: adapting to the pandemic 3. Processes: duty to be on the frontlines of care delivery
Tan et al. (28), China	To explore the work experience of clinical first-line nurses treating patients with COVID-19	Qualitative study; purposive sampling	First wave of pandemic(January to February, 2020)	Nurses (*n* = 30;average age of 31.23 ± 6.27; working unit: COVID-19 ward; working experience: 9.10 ± 5.90 years)	Semi-structured interviews; content analysis	1. Negative experiences during clinical first-line work 2. Positive impacts of clinical first-line work
Muz et al. (54), Turkey	To reveal the physical, psychological, social and professional experiences of nurses caring for COVID-19 patients	Qualitative study; Purposive sampling	First wave of pandemic(June to August, 2020)	Nurses (*n* = 19;age group: 23–40; working unit: pandemic ward, pandemic ICU; working experience: 1–18 years)	Semi-structured interview; Colaizzi's seven-step method	1. First meeting and getting caught unprepared 2. Social isolation and loneliness3. Dilemma and conflict in professional roles 4. Nursing: power emerging from difficulties 5. Organizational expectations
Moradi et al. (42), Iran	To explore the challenges experienced by ICU nurses throughout the provision of care for COVID-19 patients	Qualitative study; Purposive sampling	First wave of pandemic	Nurses (*n* = 17; age group: 27–43; working unit: medical ICUs of a COVID-19 center; working experience: 2–17 years)	Semi-structured face-to-face interview; content analysis	1. Organization's in efficiency in supporting nurses 2. Physical exhaustion 3. Living with uncertainty 4. Psychological burden of the disease
Demirci et al. (55), Turkey	To explore Turkish nurses' experiences of working at COVID-19 pandemic units	Qualitative study; Purposive sampling and theoretical sampling	First wave of pandemic(June 2020)	Nurses (*n* = 15;age group: 21–39; working unit: COVID-19 pandemic unit; working experience: 1–22 years)	in-depth telephonic interviews; constant comparative method	1. Being in the pandemic2. Empowerment for coping with the struggle 3. Challenges during the coping process 4. Effects of the pandemic on life
Fernández-Castillo et al. (69), Spain	To explore and describe the experiences and perceptions of nurses caring for COVID-19 patients in ICU	Qualitative study; homogeneous purposive sampling	First wave of pandemic(April 12 to April 30, 2020)	Nurses (*n* = 17;age group: 31–54; working unit: ICU; working experience: 3–25 years)	Semi-structured interviews; thematic analysis	1. Providing nursing care 2. Resources management and safety 3. Psychosocial aspects and emotional lability 4. Professional relationships and fellowship
Lee and Lee (16), South Korea	To explore the experiences of COVID-19-designated hospital nurses in South Korea who provided care for patients based on their lived experiences	Qualitative study; snowball sampling	First wave of pandemic (June 8 to September 25, 2020)	Nurses (*n* = 18; age group: 20–49; working unit: COVID-19 isolation ward; working experience: 7.44 ± 5.90 years)	In-depth interviews; Giorgi's phenomenological methodology	1. Pushed onto the Battlefield without Any Preparation 2. Struggling on the Frontline 3. Altered Daily Life 4. Low Morale 5. Unexpectedly Long War 6. Ambivalence Toward Patients 7. Forces That Keep Me Going 8. Giving Meaning to My Work 9. Taking Another Step in One's Growth
Danielis et al. (10), Italy	To describe the experiences of Italian nurses who have been urgently and compulsorily allocated to a newly established COVID-19sub-ICU	Qualitative study; maximum variation purposeful sampling	First wave of pandemic(March 21 to April 24, 2020)	Nurses (*n* = 24; age group: 34.1 ± 6.7; working unit: sub-ICU created for COVID-19 patients; working experience: 9.3 ± 6.8 years)	Focus group; thematic analysis	1. Becoming a frontline nurse 2. Living a double-faced professional experience 3. Advancing in nursing practice
Simşek and Günay (56), Turkey	To examine the experiences and feelings of parent nurses who care for COVID-19 patients	Qualitative study; purposeful sampling	First wave of pandemic(May to July 24, 2020)	Nurses (*n* = 26; age group: over 20; working unit: COVID-19 clinics; working experience: 1–12 years)	content analysis; written documents	1. Longing 2. Fear 3. Concern 4. Despair 5. Professional responsibility
Cui et al. (23), China	To explore the experiences and psychological adjustments of nurses who voluntarily supported COVID-19 patients	Qualitative study; purposeful sampling	First wave of pandemic (April 10 and May 7, 2020)	Nurses [*n* = 12; age group: 34.67 ± 4.10 (25–45); working unit: COVID-19 ward; working experience: 13.58 ± 4.66 years]	Semi-structured, face-to-face interviews; content analysis	1. Motivations for supporting the hardest-hit areas 2. Challenges faced during the support missions 3. Psychological experiences 4. Psychological adjustments 5. Personal and professional growth
Iheduru-Anderson et al. (62), US	To describe the lived experience of acute care nurses working with limited access to PPE during the COVID-19 pandemic.	Qualitative study; purposeful sampling	First wave of pandemic(May 15 to June 20, 2020)	Nurses (*n* = 28; age group: 28–65; working unit: medical–surgical unit, emergency department, ICU; working experience: 3–42 years)	Unstructured interviews; thematic analysis	1. Emotional roller coaster 2. Self-care habits/changed how I work 3. Hoping for the best 4. Nurses are not invincible 5. “I feel lucky”
García-Martín et al. (70), Spain	To explore the experiences and perceptions of recent nursing graduates during the COVID-19 outbreak	Qualitative study; convenience and snowball sampling	First wave of pandemic(February and April, 2020)	Nurses (*n* = 16;average age of 26.25 ± 3.30; working unit: emergency department; working experience: ≤ 6 months in emergency departments)	Semi-structured one-to-one interviews; content analysis	1. Fears and concerns 2. Organizational issues 3. Support for novice nurses
Sarnkhaowkhom et al. (79), Thailand	To explore the experiences of novice nurses caring for COVID-19 patients	Qualitative study; purposive sampling	Second wave of pandemic (December 2020 and January 2021)	Nurses (*n* = 12; age group: 22–26; working unit: COVID-19 ward; working experience: 2 months−2 years and 5 months)	In-depth interview; thematic analysis	1. From novice nurses to nurse who care for COVID-19 patients 2. Various learning methods focused on providing care to COVID-19 patients 3. Work experiences and confrontations with COVID-19 4. Various feelings that arise when being a nurse caring for patients with COVID-19 5. The power of novice nurse to bring along positive changes.
Rhéaume et al. (72), Canada	To explore the experiences of Canadian ICU nurses caring for COVID-19 patients	Qualitative study within a larger mixed-methods study; convenience sampling	Second wave of pandemic (January to March, 2021)	Nurses (*n* = 108; average age of 35.62; working unit: ICU; working experience: 10.61 years in average)	Critical incident technique; thematic analysis	1. Managing the pandemic 2. Witness to families' grief 3. Our safety 4. Futility of care.
Gunawan et al. (80), Indonesia	To explore the lived experience of nurses in combatting COVID-19	Qualitative study; purposive sampling	First wave of pandemic (March to June, 2020)	Nurses (*n* = 17; average age of 34; working unit: isolation unit; working experience: 1–5 years)	Semi-structured online interview and chat; thematic analysis	1. Feeling “nano-nano” 2. Lack of N95 masks 3. We are just pawns 4. Being rejected 5. Please do not spread our identity 6. Wemiss home 7. Feeling betrayed by regulation
Yildirim et al. (57), Turkey	To explore the experiences of the first nurses assigned to work in COVID-19 units	Qualitative study; purposive and snowball sampling	First wave of pandemic (May 27 to August 25, 2020)	Nurses (*n* = 17; average age of 28.52; working unit: COVID-19 ward; working experience: 4.5 months−20 years)	Semi-structured interview; Colaizzi's seven-step method	1. Needs 2. Anger 3. Questioning 4. Decision
Cerit and Uzun (58) Turkey	To examine the experiences of nurses working during the COVID-19 pandemic	Qualitative study; criterion sampling	First wave of pandemic (August 7 to 18, 2020)	Nurses (*n* = 9; average age of 31.78; working unit: COVID-19 clinic; working experience: 9.33 years in average)	Semi-structured, face-to-face interview; content analysis	1. Nurses' perceptions of the pandemic process 2. Nurses' feelings about the pandemic process 3. Difficulties nurses experienced in caring for patients with COVID-19 4. Professional achievements nurses gained while working in the COVID-19 clinic 5. Nursing care practices and changes during the COVID-19 pandemic
Mohammadi et al. (43), Iran	To describe the caregivers' experiences of the caring challenges in patients with COVID-19	Qualitative study; purposive sampling	First wave of pandemic (February to May, 2020)	Nurses (*n* = 23; age group: 24–52; working unit: COVID-19 ward; working experience: 1–16 years)	Semi-structured interview; Colaizzi's seven-step method	1. Psychological tension 2. Inefficient management 3. Contextual factors
Vejdani et al. (49), Iran	To determine the challenges faced by nurses while caring for COVID-19 patients	Qualitative study; purposive sampling	2020 (NA)	Nurses (*n* = 10; age group: 31–41; working unit: COVID-19 ward; working experience: 6–21 years)	Semi-structured interviews content analysis	1. Miss-management in controlling corona conditions 2. Mental and physical complications and challenges in corona work conditions 3. Lack of sufficient workforce
Sharififar et al. (45), Iran	To explore the challenges faced by nurses while caring for COVID-19 patients	Qualitative study; purposive sampling	First wave of pandemic (February 20 to April 18,2020)	Nurses (*n* = 12; average age of 31.47; working unit: COVID-19 ward; working experience: 11.42 years in average)	Semi-structured interview; thematic analysis	1. Stress and psychological issues 2. Equipment-related challenges 3. Increased error events 4. Treatment and medication problems 5. Hospital management
Xu et al. (30), China	To explore the experience of front-line nurses who participated in rescuing Wuhan	Qualitative study; purposive sampling with maximum variation strategy	First wave of pandemic (February 25 to March 5, 2020)	Nurses (*n* = 9; age group:30–38; working unit: COVID-19 ward; working experience: 7–14 years)	Semi-structured interview; content analysis	1. Worries and stress during rescue 2. Difficulties encountered during rescue 3. Experience of team work 4. Experience of interaction with COVID-19 patients 5. Experience of logistic support and widespread concern 6. Value and significance of the experience
Bahramnezhad et al. (46), Iran	To explore the lived experiences of nurses caring for COVID-19 patients	Qualitative study; purposive sampling with maximum variation strategy	First wave of pandemic (March 10 to May 5,2020)	Nurses (*n* = 14; average age of 31.24 ± 6.64; working unit: COVID-19 ward; working experience: 9.88 ± 6.03 years)	One-to-one in-depth recorded interview; Van Manen's approach	1. Strong pressure because of coronavirus 2. Turn threats into opportunities 3. Nurses' expectations
Lulgjuraj et al. (63), USA	To explore the experiences of pediatric nurse caring for adult COVID-19 patients	Qualitative study; purposive sampling	Second wave of pandemic (December 2020 to January2021)	Nurses (*n* = 81; age group: 20–61; working unit: COVID-19 ward; working experience: 0–21+ years)	Descriptive survey; thematic analysis.	1. Concerns for safety 2. Unprepared to care 3. Nurses' emotional responses 4. Persevering together
Chegini et al. (47), Iran	To explore the experiences of critical care nurses caring for COVID-19 patients	Qualitative study; purposive and snowball sampling	First wave of pandemic (May to June,2020)	Nurses (*n* = 15; age group: 28–50; working unit: critical care unit; working experience: 2–35 years)	Semi-structured, in-depth interview; Colaizzi's seven-step method	1. Psychological challenges 2. Organizational challenges 3. Social challenges 4. Professional challenges
Conz et al. (74), Brazil	To explore the experiences of ICU nurses caring for COVID-19 patients	Qualitative study; snowball sampling	First wave of pandemic (July to September,2020)	Nurses (*n* = 20; age group: 28–54; working unit: ICU; working experience: 2–31 years)	Online phenomenological interview; according to a theoretical study and needs of the present study	1. Adjusting to the new way of delivering care in intensive care units 2. Being around situations that interfere with physical and mental health 3. Projecting professional life after the COVID-19 pandemic
Akkuş et al. (59), Turkey	To identify the experiences and challenges faced by nurses working in COVID-19 wards	Qualitative study; snowball sampling	First wave of pandemic (May to September, 2020)	Nurses (*n* = 19; average age of 31.9 ± 7.2; working unit: COVID-19 ward; working experience: 9.9 ± 8.3 years)	Semi-structured interview; thematic analysis	1. Psychosocial adaptation 2. Protection 3. Difficulty in care and treatment 4. Access to information 5. Working conditions
Ahmadidarrehsima et al. (48), Iran	To explore the experiences of nurses caring for COVID-19 patients	Qualitative study; purposive sampling	Second wave of pandemic (December 2020 to February2021)	Nurses (*n* = 10; age group:25–44; working unit: COVID-19 care unit; working experience: 3–15 years)	Semi-structured interview; content analysis	1. Physical, psychological, and social burden of care 2. Unmet needs 3. Positive experiences 4. Strategies
Specht et al. (81), Denmark	To explore the experiences of nurse working in a newly organized COVID-19 ward with high-risk patients	Qualitative study; purposive sampling with maximum variation strategy	First wave of pandemic (June to July, 2020)	Nurses (*n* = 23; age group: 26–54; working unit: COVID-19 ward; working experience: 0.5–27 years)	Semi-structured interview; Paul Ricoeur's theory of narrative and interpretation	1. Challenging and uncertain situation but also a positive experience 2. Professional and personal development 3. Lack of nurses' rights during a pandemic 4. Reward in itself or a desire for financial reward
Cadge et al. (64), USA	To explore the experiences of nurse caring for COVID-19 patients in ICU	Qualitative study; purposive sampling	First wave of pandemic(June to August, 2020)	Nurses (*n* = 14; average age of 34.3 ± 9.6; working unit: COVID-19 ward; working experience: 10.9 ± 7.9 years)	Semi-structured interview; deductive and inductive strategies	1. Challenges of working with new co-workers and teams 2. Challenges of maintaining existing working relationships 3. Role of nursing leadership in providing information and maintaining morale 4. The importance of institutional-level acknowledgment of their work
Mohammed et al. (73), Canada	To explore the lived experiences of Canadian front-line medicine nurses caring for COVID-19 patients	Qualitative study; purposive sampling	First wave of pandemic(March to July, 2020)	Nurses (*n* = 43; age group: NA; working unit: COVID-19 ward; working experience: NA)	Semi-structured interview; Diekelmann's method	1. A traumatic experience 2. Living through the experience 3. Achieving transcendence
Moghaddam-Tabrizi and Sodeify (49), Iran	To explore the lived experiences of nurses caring for COVID-19 patients	Qualitative study; purposive sampling with maximum variation strategy	First wave of pandemic(March to May, 2020)	Nurses (*n* = 14; age group: 22–43; working unit: COVID-19 ward; working experience: 6 years in average)	Semi-structured interview; Dickelman's seven-step method	1. Staying in an ethical dilemma 2. Emotional turmoil 3. Response to professional commitments 4. Seeking help
Khanjariana and Sadat-Hoseini (50), Iran	To explore the lived experiences of nurses providing altruistic care for COVID-19 patients	Qualitative study; purposive sampling	First wave of pandemic (Spring, 2020)	Nurses (*n* = 12; age group: 25–44; working unit: COVID-19 ward; working experience: 1–18 years)	Semi-structured interview; Glaizer technique	1. Disquietude 2. Intellectuality 3. Human Transcendence
Pogoy and Cutamora (77), Philippines	To explore the experiences of Overseas Filipino Worker nurses working inCOVID-19 ICU	Qualitative study; purposive sampling	2020(NA)	Nurses (*n* = 8; age group: 28–32; working unit: COVID-19 ICU; working experience: NA)	Semi-structured interview; thematic Analysis	1. Challenges During the Pandemic 2. Patient Care During COVID-19 3. Adapting to Change 4. Resilience Amidst the Pandemic
Peng et al. (31), China	To explore the experience of frontline nurses fighting against the COVID-19 pandemic	Qualitative study; convenience sampling	First wave of pandemic (March 2020)	Nurses (*n* = 20; age group: 24–43; working unit: COVID-19 ward; working experience: 3–25 years)	Semi-structured interview; thematic Analysis	1. Negative experience 2. Positive experience
Ji and Lee (71), South Korea	To explore the experiences of new nurses caring for COVID-19 patients	Qualitative study; snowball sampling	First wave of pandemic (September to November, 2020)	Nurses (*n* = 9; age group: 24–27; working unit: COVID-19 ward; working experience: <1 to 2 years)	Unstructured interview; Colaizzi's method	1. Fear as a new nurse who has not experienced infectious disease 2. Physical and psychological burden in isolation environment 3. Building professional values
Naylor et al. (65), USA	To describe the experience of novice nurses working in acute care settings during a pandemic	Qualitative study; snowball sampling	NA	Nurses (*n* = 13; age group: 24–41; working unit: acute care setting for COVID-19 patients; working experience: 6–18 months)	Semi-structured interview; thematic Analysis	1. Dealing with death 2. Which personal protective equipment (PPE)will keep us safe 3. Caring for high acuity patients with limited training 4. Difficulties working short staffed 5. Everything is not okay 6. Support from the healthcare team 7. Nursing school preparation for a pandemic 8. I would still choose nursing
Chau et al. (32), China	To explore the experiences of nurses caring for patients with suspected or diagnosed COVID-19	Qualitative study; purposive sampling	First wave of pandemic (June to August, 2020)	Nurses (*n* = 39; age group: 20–59; working unit: COVID-19 ward; working experience: 1–15+ years)	Semi-structured interview; thematic Analysis	1. Confronting resource shortages 2. Changes in usual nursing responsibilities and care modes 3. Maintaining physical and mental health 4. Need for effective and timely responses from relevant local authorities 5. Role of the community in public health protection and management 6. Advanced pandemic preparedness
Hu et al. (33), China	To examine ICU nurses' experiences of caring for patients with COVID-19	Qualitative study; purposive sampling	First wave of pandemic (April to May, 2020)	Nurses (*n* = 13; age group: 24–38; working unit: ICU; working experience: 1–13 years)	Individual interview; Colaizzi's seven-step method	1. Stage 1: initial response 2. Stage 2: adaption 3. Stage 3: desperation 4. Stage 4: holding on and surviving
Rathnayake et al. (84), Sri Lanka	To explore the experiences and challenges of nurses caring for COVID-19 patients	Qualitative study; purposive and snowball sampling	First wave of pandemic (June 2020)	Nurses (*n* = 14; average age of33.36 ± 6.3; working unit: COVID-19 ward; working experience:8.14 ± 6.11 years)	In-depth telephone interview; Colaizzi's method	1. Physical and psychological distress among nurses 2. Willingness to work 3. Educational and information needs of nurses 4. Essential role in support mechanisms 5. Modern technology in COVID-19 care
Zamanzadeh et al. (51), Iran	To identify nurses' experiences in caring for COVID-19 patients	Qualitative study; purposive sampling	First wave of pandemic (April to October, 2020)	Nurses (*n* = 20; age group:25–49; working unit: ICU, Emergency, Internal medicine; working experience:2–26 years)	Semi-structured interview; content analysis	1. Duality in the form of care 2. Confusion and ambiguity in care planning 3. Workload 4. Social isolation in spite of positive image
Çakici et al. (60), Turkey	To identify the challenges experienced by nurses caring for COVID-19 patients	Qualitative study; maximum variation sampling	First wave of pandemic (March to April, 2020)	Nurses (*n* = 15; average age of26.53 ± 3.52; working unit: COVID-19 ward; working experience:4.53 ± 2.82 years)	Semi-structured in-depth interview; descriptive analysis	1. Concern and fear of being infected 2. Change in the family order 3. Performing patient care with fear 4. Social stigma 5. Questioning the nurse's role within the health system 6. Difficulty working with personal protective equipment 7. Physical injury caused by equipment
Yip et al. (34), China	To examine the experiences of junior nurses caring for COVID-19 patients	Qualitative study; purposive sampling	Second wave of pandemic (January to May, 2021)	Nurses (*n* = 40; age group: 21–35; working unit: isolation ward; working experience: 2–4 years)	Individual interviews; Colaizzi's seven-step method	1. Hurdles in the Early Stage 2. Self-Care Coping Strategies 3. Staying Positive under Pressure 4. Perceived Negativity and Positivity: Two Sides of Emotions
Li et al. (35), China	To discuss the work experience of front-line nurses involving COVID-19 rescue	Qualitative study; NA	First wave of pandemic (January to February, 2020)	Nurses (*n* = 23; average age of 31.48 ± 2.30; working unit: COVID-19 ward; working experience: 1–30 years)	Semi-structured interview; Colaizzi's method	1. They had different emotional experiences during the aiding period 2. Aiding work had a double impact on the nurses 3. There were certain difficulties in aiding work 4. There were significant age differences in aiding work experience
Villar et al. (83), Qatar	To explore the lived experiences of frontline nurses caring for COVID-19 patients	Qualitative study; purposive and snowball sampling	First wave of pandemic (September to October, 2020)	Nurses (*n* = 30; average age of 31 ± 2.8; working unit: critical care unit, inpatient, emergency; working experience: 9.1 ± 2.7yaers)	Semi-structured interview; Colaizzi's method	1. Challenges of working in a COVID-19 facility 2. Surviving COVID-19 3. Resilience of Nurses
Chen et al. (36), China	To explore the experiences of frontline nurses during the COVID-19 pandemic	Qualitative study; purposive sampling	First wave of pandemic (January to April, 2020)	Nurses (*n* = 12; age group: NA; working unit: COVID-19 ward; working experience: NA)	Diaries written by certified frontline nurses from social media; content analysis	1. Constructing a better self 2. Constructing a strong support network
Gordon et al. (66), USA	To explore the experiences of critical care nurse caring for COVID-19 patients	Qualitative study; purposive sampling	NA	Nurses (*n* = 11; age group: 23–60; working unit: ICU; working experience: 7.9 ± 7.8,7.2 ± 7.6 and 3.6 ± 2.8 years in practice, ICU and current unit)	Semi-structured interview; content analysis	1. Emotions experienced 2. Physical symptoms 3. Care environment challenges 4. Social effects 5. Short term coping strategies
Sadang (76), Filipino	To explore the lived experience of nurses working in COVID-19 quarantine facilities	Qualitative study; purposive and snowball sampling	NA	Nurses (*n* = 12; age group: 25–38; working unit: quarantine facility; working experience: NA)	Individual in-depth interviews; content analysis	1. Work as self-sacrifice 2. Work as self-fulfillment 3. Work as a psychological struggle
Robinson and Stinson (67), USA	To explore the experiences of nurse caring for COVID-19 patients	Qualitative study; purposive and snowball sampling	NA	Nurses (*n* = 14; age group: 25–59; working unit: ICU, emergency, medical-surgical unit; working experience: 1–33 years)	Semi-structured interview; Colaizzi's method	1. The Human Connection 2. The nursing burden 3. Coping
Zhou et al. (37), China	To explore the experience of newly recruited male nurses during the COVID-19 pandemic	Qualitative study; purposive sampling	First wave of pandemic (March 2020)	Nurses (*n* = 9; average age of 25 ± 2.0; working unit: COVID-19 ward; working experience: NA)	Semi-structured interview; Colaizzi's seven- step method	1. Impact of the epidemic 2. Gain experience and growth in the fight against the epidemic 3. Need for nurses in the epidemic
Queiroz et al. (75), Brazil	To explore nurses' experiences and feelings during the COVID-19pandemic	Qualitative study; snowball sampling	First wave of pandemic (April to June, 2020)	Nursing professionals(*n* = 719; NA; working unit: COVID-19 ward; working experience: 14 years in average)	Questionnaire; the Discourse of the Collective Subject, Symbolic Interactionism	1. Interaction with the ‘new' 2. Interaction with nursing care 3. Interaction with daily work
Jia et al. (38), China	To examine the ethical challenges encountered by nurses caring for patients with COVID-19	Qualitative study; purposive sampling	First wave of pandemic (February to March, 2020)	Nurses (*n* = 18; age group: 24–43; working unit: COVID-19 ward; working experience: 2–33 years)	Structured in-depth interviews; content analysis	1. Ethical challenges 2. Coping styles 3. Impacts on career
Rezaee et al. (52), Iran	To explore the nurses' perception of ethical challenges during the COVID-19 pandemic	Qualitative study; purposive sampling	First wave of pandemic (September to October, 2020)	Nurses (*n* = 24; age group: 27–49 years; working unit: COVID-19 ward; working experience: 4–16 years)	Structured in-depth interviews; content analysis	1. Threats to professional values 2. The absence of a holistic COVID-19 care approach
Gao et al. (39), China	To explore nurses' experiences regarding shift patterns while providing front line care for COVID 19 patients in isolation wards of hospitals	Qualitative study; purposive sampling	First wave of pandemic (2020)	Nurses (*n* = 14; average age of 33.5 ± 6. 0; working unit: isolation ward; working experience: 2–23 years)	Semi structured in depth interviews based; Colaizzi's method	1. Assess the competency of nurses to assign nursing work scientifically and reasonably 2. Reorganize nursing workflow to optimize shift patterns 3. Communication between managers and front line nurses to humanize shift patterns 4. Nurses' various feelings and views on shift patterns
Fawaz and Itani (82), Lebanon	To explore the psychological experiences of Lebanese frontline nurses serving at ground zero hospital during the COVID-19 outbreak	Qualitative study; purposive sampling	Second wave of pandemic (January 2021)	Nurses (*n* = 18; average age of 24. 6; working unit: COVID-19 ward, ICU, COVID-19 emergency departments; working experience: at least 1 years)	Virtual interviews; thematic analysis	1. Helplessness and impending doom 2. Increased mortality rates and depressive mood 3. Fear of death and obsessive thinking 4. Public recklessness, governmental responsibility, and anger 5. Flashbacks, panic, and incompetence

### Quality appraisal

According to CASP criteria, the 70 included studies had good quality by clearly describing at least 8 of the 10 items. All of the studies had a clear statement of their aims and findings. All of the studies used appropriate qualitative methodology, research design, data collection, and data analysis. The majority of the studies clearly stated recruitment strategies and took ethical issues into consideration. Only eleven studies considered the relationship between the researcher and the participants. No study was excluded, and all details of quality assessment are provided in Table 3.

**Table 3:** Quality assessment of included studies.

**Included study**	**1**	**2**	**3**	**4**	**5**	**6**	**7**	**8**	**9**	**10**
Sun et al. (26)	Y	Y	Y	Y	Y	Y	Y	Y	Y	Valuable
Liu et al. (22)	Y	Y	Y	Y	Y	Y	Y	Y	Y	Valuable
Zhang et al. (27)	Y	Y	Y	Y	Y	C	Y	Y	Y	Valuable
Fan et al. (24)	Y	Y	Y	Y	Y	C	Y	Y	Y	Valuable
Liu et al. (25)	Y	Y	Y	Y	Y	C	Y	Y	Y	Valuable
Catania et al. (14)	Y	Y	Y	C	Y	C	Y	Y	Y	Valuable
Kackin et al. (53)	Y	Y	Y	Y	Y	C	Y	Y	Y	Valuable
Alizadeh et al. (12)	Y	Y	Y	Y	Y	C	Y	Y	Y	Valuable
Sadati et al. (11)	Y	Y	Y	Y	Y	C	Y	Y	Y	Valuable
Galehdar et al. (40)	Y	Y	Y	Y	Y	C	Y	Y	Y	Valuable
He et al. (13)	Y	Y	Y	Y	Y	C	C	Y	Y	Valuable
Karimi et al. (9)	Y	Y	Y	Y	Y	C	Y	Y	Y	Valuable
García-Martín et al. (70)	Y	Y	Y	Y	Y	C	Y	Y	Y	Valuable
Schroeder et al. (61)	Y	Y	Y	Y	Y	C	Y	Y	Y	Valuable
Galehdar et al. (41)	Y	Y	Y	Y	Y	C	Y	Y	Y	Valuable
Tan et al. (28)	Y	Y	Y	Y	Y	C	Y	Y	Y	Valuable
Yin and Zeng (29)	Y	Y	Y	Y	Y	C	Y	Y	Y	Valuable
Arcadi et al. (68)	Y	Y	Y	Y	Y	C	Y	Y	Y	Valuable
Ohta et al. (78)	Y	Y	Y	Y	Y	Y	Y	Y	Y	Valuable
Muz and Yüce (54)	Y	Y	Y	Y	Y	C	Y	Y	Y	Valuable
Moradi et al. (42)	Y	Y	Y	Y	Y	C	Y	Y	Y	Valuable
Demirci et al. (55)	Y	Y	Y	Y	Y	Y	Y	Y	Y	Valuable
Fernández-Castillo et al. (69)	Y	Y	Y	Y	Y	C	Y	Y	Y	Valuable
Lee and Lee (16)	Y	Y	Y	Y	Y	C	Y	Y	Y	Valuable
Danielis et al. (10)	Y	Y	Y	Y	Y	Y	Y	Y	Y	Valuable
Simşek and Günay (56)	Y	Y	Y	Y	Y	C	Y	Y	Y	Valuable
Iheduru-Anderson (62)	Y	Y	Y	Y	Y	C	Y	Y	Y	Valuable
Cui et al. (23)	Y	Y	Y	Y	Y	C	Y	Y	Y	Valuable
Sarnkhaowkhom et al. (79)	Y	Y	Y	Y	Y	C	Y	Y	Y	Valuable
Rhéaume et al. (72)	Y	Y	Y	Y	Y	C	Y	Y	Y	Valuable
Gunawan et al. (80)	Y	Y	Y	Y	Y	C	Y	Y	Y	Valuable
Yildirim et al. (57)	Y	Y	Y	Y	Y	C	Y	Y	Y	Valuable
Cerit and Uzun (58)	Y	Y	Y	Y	Y	C	Y	Y	Y	Valuable
Mohammadi et al. (43)	Y	Y	Y	Y	Y	C	Y	Y	Y	Valuable
Vejdani et al. (44)	Y	Y	Y	Y	Y	C	Y	Y	Y	Valuable
Sharififar et al. (45)	Y	Y	Y	Y	Y	C	Y	Y	Y	Valuable
Xu et al. (30)	Y	Y	Y	Y	Y	C	Y	Y	Y	Valuable
Bahramnezhad et al. (46)	Y	Y	Y	Y	Y	C	Y	Y	Y	Valuable
Lulgjuraj et al. (63)	Y	Y	Y	Y	Y	C	Y	Y	Y	Valuable
Chegini et al. (47)	Y	Y	Y	Y	Y	C	Y	Y	Y	Valuable
Conz et al. (74)	Y	Y	Y	Y	Y	C	Y	Y	Y	Valuable
Akkuş et al. (59)	Y	Y	Y	Y	Y	C	Y	Y	Y	Valuable
Ahmadidarrehsima et al. (48)	Y	Y	Y	Y	Y	C	Y	Y	Y	Valuable
Specht et al. (81)	Y	Y	Y	Y	Y	Y	Y	Y	Y	Valuable
Cadge et al. (64)	Y	Y	Y	Y	Y	C	Y	Y	Y	Valuable
Mohammed et al. (73)	Y	Y	Y	Y	Y	Y	Y	Y	Y	Valuable
Moghaddam-Tabrizi and Sodeify (49)	Y	Y	Y	Y	Y	C	Y	Y	Y	Valuable
Khanjariana and Sadat-Hoseini (50)	Y	Y	Y	Y	Y	C	Y	Y	Y	Valuable
Pogoy and Cutamora (77)	Y	Y	Y	Y	Y	C	Y	Y	Y	Valuable
Peng et al. (31)	Y	Y	Y	Y	Y	C	Y	Y	Y	Valuable
Ji and Lee (71)	Y	Y	Y	Y	Y	C	Y	Y	Y	Valuable
Naylor et al. (65)	Y	Y	Y	Y	Y	Y	Y	Y	Y	Valuable
Chau et al. (32)	Y	Y	Y	Y	Y	C	Y	Y	Y	Valuable
Hu et al. (33)	Y	Y	Y	Y	Y	C	Y	Y	Y	Valuable
Rathnayake et al. (84)	Y	Y	Y	Y	Y	C	Y	Y	Y	Valuable
Zamanzadeh et al. (51)	Y	Y	Y	Y	Y	C	Y	Y	Y	Valuable
Çakici et al. (60)	Y	Y	Y	Y	Y	C	Y	Y	Y	Valuable
Yip et al. (34)	Y	Y	Y	Y	Y	C	Y	Y	Y	Valuable
Li et al. (35)	Y	Y	Y	C	Y	Y	C	C	Y	Valuable
Villar et al. (83)	Y	Y	Y	Y	Y	C	Y	Y	Y	Valuable
Chen et al. (36)	Y	Y	Y	Y	Y	C	Y	Y	Y	Valuable
Gordon et al. (66)	Y	Y	Y	Y	Y	C	Y	Y	Y	Valuable
Sadang (76)	Y	Y	Y	Y	Y	C	Y	Y	Y	Valuable
Robinson and Stinson (67)	Y	Y	Y	Y	Y	C	Y	Y	Y	Valuable
Zhou et al. (37)	Y	Y	Y	Y	Y	Y	Y	Y	Y	Valuable
Queiroz et al. (75)	Y	Y	Y	Y	Y	C	Y	Y	Y	Valuable
Jia et al. (38)	Y	Y	Y	Y	Y	C	Y	Y	Y	Valuable
Gao et al. (39)	Y	Y	Y	Y	Y	Y	Y	Y	Y	Valuable
Fawaz and Itani (82)	Y	Y	Y	Y	Y	C	Y	Y	Y	Valuable
Rezaee et al. (52)	Y	Y	Y	Y	Y	C	Y	Y	Y	Valuable

1. Was there a clear statement of the aims of the research?

2. Is a qualitative methodology appropriate?

3. Was the research design appropriate to address the aims of the research?

4. Was the recruitment strategy appropriate for the aims of the research?

5. Were the data collected in a way that addressed the research issue?

6. Has the relationship between researcher and participants been adequately considered?

7. Have ethical issues been taken into consideration?

8. Was the data analysis sufficiently rigorous?

9. Is there a clear statement of findings?

10. How valuable is the research?

Y, Yes; C, Cannot Tell; N, No.

### Meta-synthesis of qualitative data

Two hundred eighty-five findings were extracted from the included studies, and were classified as Unequivocal or Credible. From a meta-synthesis analysis of the data, five synthesized findings were developed from 26 categories (see Table 4).

**Table 4:** Meta-synthesis of findings.

**Synthesized finding**	**Synthesized finding 1**				**Synthesized finding 2**								**Synthesized finding 3**					**Synthesized finding 4**						**Synthesized finding 5**		
**Category**	**1**	**2**	**3**	**4**	**5**	**6**	**7**	**8**	**9**	**10**	**11**	**12**	**13**	**14**	**15**	**16**	**17**	**18**	**19**	**20**	**21**	**22**	**23**	**24**	**25**	**26**
Sun et al. (26)		✓		✓									✓		✓									✓		✓
Liu et al. (22)	✓			✓	✓			✓	✓	✓			✓	✓	✓									✓		
Zhang et al. (27)	✓	✓	✓	✓				✓																		
Fan et al. (24)	✓	✓			✓				✓																	
Liu et al. (25)	✓		✓	✓				✓		✓									✓				✓			
Catania et al. (14)		✓	✓		✓	✓		✓	✓	✓					✓	✓								✓		
Kackin et al. (53)		✓	✓			✓		✓	✓				✓					✓		✓						
Alizadeh et al. (12)			✓			✓		✓	✓		✓	✓	✓	✓	✓					✓						
Sadati et al. (11)	✓	✓				✓		✓					✓													
He et al. (13)	✓	✓	✓	✓	✓			✓																		
Galehdar et al. (40)		✓	✓				✓	✓			✓															
Galehdar et al. (41)						✓		✓	✓												✓	✓		✓	✓	
Karimi et al. (9)		✓	✓			✓		✓											✓	✓						
García-Martín et al. (70)			✓		✓											✓			✓							
Schroeder et al. (61)	✓								✓			✓	✓		✓	✓										
Tan et al. (28)		✓	✓					✓	✓	✓								✓	✓	✓				✓		
Yin and Zeng (29)						✓					✓							✓	✓	✓		✓				
Arcadi et al. (68)		✓	✓		✓		✓	✓																✓	✓	
Ohta et al. (78)		✓	✓	✓		✓							✓		✓				✓							
Muz and Yüce (54)		✓				✓	✓	✓													✓	✓		✓	✓	
Moradi et al. (42)		✓	✓			✓		✓	✓												✓					
Demirci et al. (55)			✓			✓							✓	✓										✓	✓	✓
Fernández-Castillo et al. (69)		✓			✓				✓										✓	✓		✓		✓		
Lee and Lee (16)		✓	✓	✓	✓	✓		✓	✓								✓	✓	✓	✓						
Danielis et al. (10)		✓			✓											✓								✓		
Simşek and Günay (56)	✓		✓			✓	✓	✓																		
Cui et al. (23)	✓	✓	✓		✓	✓		✓		✓			✓	✓	✓									✓	✓	✓
Iheduru-Anderson (62)			✓	✓					✓				✓							✓		✓				
Sarnkhaowkhom et al. (79)	✓	✓	✓		✓	✓		✓		✓				✓		✓			✓							
Rhéaume et al. (72)			✓				✓	✓	✓	✓		✓					✓		✓	✓		✓				
Gunawan et al. (80)	✓	✓				✓		✓			✓											✓				
Yildirim et al. (57)		✓				✓			✓											✓	✓	✓		✓		✓
Cerit and Uzun (58)		✓		✓	✓	✓		✓	✓								✓							✓		
Mohammadi et al. (43)		✓	✓					✓	✓		✓	✓				✓										
Vejdani et al. (44)		✓	✓					✓			✓	✓				✓		✓			✓					
Sharififar et al. (45)		✓				✓		✓	✓									✓	✓							
Xu et al. (30)		✓	✓	✓		✓		✓	✓			✓	✓											✓		✓
Bahramnezhad et al. (46)		✓			✓	✓	✓	✓	✓		✓										✓			✓	✓	
Lulgjuraj et al. (63)		✓	✓	✓				✓	✓				✓		✓	✓		✓	✓							
Chegini et al. (47)		✓		✓		✓		✓	✓		✓	✓						✓	✓	✓	✓					
Conz et al. (74)			✓	✓			✓	✓	✓	✓			✓	✓		✓	✓				✓			✓		
Akkuş et al. (59)	✓	✓	✓	✓	✓	✓	✓	✓	✓	✓		✓	✓	✓	✓			✓	✓		✓	✓			✓	
Ahmadidarrehsima et al. (48)		✓	✓	✓		✓		✓	✓				✓	✓	✓			✓		✓	✓	✓				
Specht et al. (81)		✓		✓				✓	✓	✓			✓		✓				✓		✓	✓		✓	✓	✓
Cadge et al. (64)						✓			✓						✓	✓					✓	✓			✓	
Mohammed et al. (73)		✓	✓	✓	✓			✓		✓			✓				✓					✓		✓		
Moghaddam-Tabrizi and Sodeify (49)		✓	✓		✓	✓		✓				✓									✓	✓				
Khanjariana and Sadat-Hoseini (50)	✓	✓	✓	✓	✓	✓		✓					✓		✓									✓	✓	✓
Pogoy and Cutamora (77)		✓			✓	✓	✓	✓					✓	✓										✓	✓	✓
Peng et al. (31)		✓	✓						✓															✓	✓	✓
Ji and Lee (71)		✓	✓		✓			✓	✓	✓					✓				✓	✓	✓			✓		
Naylor et al. (65)		✓				✓			✓	✓			✓		✓				✓					✓		
Chau et al. (32)				✓	✓	✓		✓			✓		✓	✓	✓	✓	✓		✓	✓		✓	✓			
Hu et al. (33)	✓	✓	✓	✓				✓		✓					✓			✓							✓	
Rathnayake et al. (84)	✓	✓	✓		✓	✓	✓	✓	✓	✓			✓	✓	✓	✓	✓	✓	✓	✓	✓	✓		✓	✓	
Zamanzadeh et al. (51)	✓		✓		✓	✓		✓	✓		✓	✓														
Çakici et al. (60)		✓	✓		✓	✓		✓			✓							✓			✓					
Yip et al. (34)		✓	✓	✓		✓		✓	✓	✓			✓	✓	✓									✓		
Li et al. (35)		✓						✓	✓															✓		
Villar et al. (83)		✓	✓	✓	✓	✓	✓						✓	✓	✓											
Chen et al. (36)				✓		✓							✓		✓									✓		✓
Gordon et al. (66)		✓	✓	✓	✓	✓		✓	✓	✓		✓	✓		✓											
Sadang (76)		✓	✓			✓		✓	✓															✓		
Robinson and Stinson (67)						✓	✓	✓	✓	✓			✓		✓	✓								✓		✓
Zhou et al. (37)		✓	✓	✓				✓	✓				✓					✓	✓			✓		✓		
Queiroz et al. (75)		✓	✓	✓	✓	✓		✓	✓					✓				✓			✓					
Jia et al. (38)			✓		✓		✓			✓			✓	✓	✓											
Gao et al. (39)																				✓						
Fawaz and Itani (82)			✓								✓															
Rezaee et al. (52)						✓	✓																			

Synthesized finding 1: Although nurses actively devoted themselves to fighting against COVID-19, considering their professional responsibility and historical previous experience with mankind, they were not invulnerable.

Synthesized finding 2: There were various difficulties and challenges in caring for patients with COVID-19, including fear related to providing patients with care, shortage of protective equipment and manpower, and negative attitude of family members.

Synthesized finding 3: Facing difficulties and challenges, nurses could only partly cope by using mixed means to overcome those, including media, learning, gaining skills, responding together, and organizational assistance.

Synthesized finding 4: To better respond to the COVID-19 pandemic, nurses' needs should be paid attention to. Counseling, training, information, resources, and investment are pivotal.

Synthesized finding 5: Despite the hardships, nurses became stronger and gained gratitude, positivity, mental peace, and confidence.

Category 1: Fighting on the front line with the spirit of Nightingale.

Category 2: Early psychological experience.

Category 3: Negative psychology peaked in the process of adapting to work.

Category 4: Being positive gradually.

Category 5: Nursing care during the COVID-19 pandemic.

Category 6: Negative social experience.

Category 7: Ethical issues in caring for patients with COVID-19.

Category 8: Challenges related to PPE.

Category 9: Challenges related to the staff shortage and workload.

Category 10: Limited knowledge and skills of nurses.

Category 11: Insufficient public education.

Category 12: Inefficient management of healthcare system.

Category 13: Nurses' individual psychological coping styles.

Category 14: Active learning through various ways.

Category 15: Seeking external support.

Category 16: Organization's coping strategies.

Category 17: Application of modern technology to caring for patients with COVID-19.

Category 18: The need for psychological support.

Category 19: The need for training and information.

Category 20: The need for resources management.

Category 21: The needs for more investments in nursing.

Category 22: The needs for the rights to be respected.

Category 23: The needs for improved response efficiency of healthcare system.

Category 24: Advancing in the nursing career.

Category 25: Improved relationships and social recognition.

Category 26: Being more positive toward life.

Synthesized finding 1: Although nurses actively devoted themselves to fighting against COVID-19, considering their professional responsibility and historical previous experience with mankind, they were not invulnerable.

Category 1: Fighting on the front-line with the spirit of Nightingale.

During the COVID-19 pandemic, nurses showed a strong sense of responsibility, and they were proud to provide nursing care for patients with COVID-19 (11, 13, 22–25, 27, 33, 50, 56, 59, 61, 79, 80, 84). Several nurses reported that their family did not want them to work at risk, but they strived for the support of family (23).

“*I am a nurse, [and] nurses were needed there [Hubei Province]. There was not much to think about. It was my passion for my profession, and a sense of responsibility.... I really wanted to go. I hoped I could do something to help others, so I signed up.” (N1, P1138)* *(**23**)*

“*As a Communist party member, in the face of this kind of emergency crisis, it was natural to have the courage to go to the front-line; what's more, our duty was lifesaving; this task was our responsibility and mission.”(N2, P761)* *(**25**)*

Despite being aware of the shortage of facilities and PPE, nurses were willing to take care of patients with COVID-19 (51). Even novice nurses volunteered to care for the patients with COVID-19 because they thought that they were young and did not have the burden of family responsibilities like senior nurses (79). A few nurses were in a dilemma of staying with family or fighting on the front-line (50). However, most nurses stated that they would stick to the front line and never back down, even if the workload was heavy and there was a risk of infection (13, 22, 51, 56).

“*Our masks and protective clothing are, indeed, nonstandard, but this is not a reason to abandon patients. I go to the patients and feed them, give them their medicine on time, and I am not worried about getting infected...”(N6, P6)* *(**51**)*

“*There is a big shortage of nurses, so I volunteered to go because I was young.” (N4, P5)* *(**79**)*

“*This is my duty because I am a medical worker. No matter what will happen…” (N3, Pe792)* *(**22**)*

Category 2: Early psychological experience.

Due to the urgent recruitment, nurses did not prepare well for the emergency task, and they feared about the uncertainty and possibility of infection in the early stage (10, 16, 23, 27, 30, 31, 33, 35, 37, 43–45, 47–50, 54, 57–60, 63, 65, 66, 68, 71, 73, 75–81, 83, 84). Many nurses worried about how to do (31, 50, 63, 81) and experienced self-doubt because taking care of patients with COVID-19 might be different from nurses' previous work (14, 16, 34, 37, 71, 75, 81). Due to fear and anxiety, some staff refused to provide care and treatment for patients with COVID-19 (31, 51). The new workplace, the different nursing routines, the nature of the disease, and inexperience made nurses feel anxiety and stressful (9, 24, 27, 28, 30, 33, 40, 42, 57, 58, 63, 69, 71, 73). The newly formed work team also led to nurses' emotional stress (13, 14, 53). Some started to worry about the health condition of their family and fear of infecting family and others (27, 37, 43–48, 50, 51, 58, 66, 71, 73, 76, 83). The unknown disease made the nurses feel stressful, and they were also concerned about the patients' condition (11, 26, 51, 58, 63). The news about COVID-19 intensified the nurses' negative psychological experience in the initial days (11, 27, 51, 65, 73). These negative psychological experiences could distract the nurses from nursing work (51).

“*When went to the ward first time, we were afraid to enter the room to contact patients, only standing in the corridor.” (N1, P4)* *(**30**)*

“*We only knew that we were going to Huangshi, we did not know to which specific hospital. Actually, we did not understand what lay ahead. Uncertainty made us psychologically uneasy.”(N1, P1138)* *(**23**)*

“*I don't know about infectious diseases, and I encountered it for the first time, so what should I do...” (N2, P4)* *(**71**)*

“*On the first day of work, when I found out that my patient had coronavirus, after going back home, I cried all my off day, lest I get sick and transfer the virus to my family. Because I really didn't know much about it, and I thought I would get infected if I came to direct contact with the patient.” (Pe85)* *(**46**)*

Category 3: Negative psychology peaked in the process of adapting to work.

Novice nurses were under great pressure due to lack of experience, and they were afraid of becoming a burden (70, 79). When they had to deal with everything on their own, they felt helpless and devastated (71, 79). The fear of being infected and the fear of infecting family and others still persisted among nurses (9, 14, 23, 25, 27, 33, 34, 40, 42, 56, 59, 60, 63, 70, 74, 75, 78, 84), so some of them checked that PPE was used in a correct way repeatedly (27) and hoped not to be infected (62). Some even questioned the effectiveness of PPE (71, 75, 84) and felt as if infected with the slightest symptom (16, 25). The COVID-19 pandemic broke out rapidly, and some hospitals were caught unprepared. Therefore, the nurses in those hospitals felt angry and abandoned due to a shortage of PPE (11, 62, 68). Due to the shortage of PPE, the nurses emphasized that they did not feel equipped physically and felt unsafe (63).

“*I went into the COVID-19 patient's room by myself …I had to go by myself because going in with other people would have cost the hospital too many PPE sets. I had to drill to do it alone by myself and complete my responsibilities since I did not want to be a burden to the team.” (N10, P7)* *(**79**)*

“*The other big fear is bringing the virus home and infecting the people you care about, which is why I've been self-isolated [speaks with a trembling voice] and decided to rent a house and go live alone where I am now.” (N2, P5)* *(**40**)*

“*I was a little scared that it would happento me, but I scared, I would give this terrible disease to my husband, then to his family, the mother is too old...” (N6, P4)* *(**84**)*

It was found that the nurses felt unfair in work distribution or awards compared with other medical staff because they contacted patients more frequent than others (16, 38, 44, 53, 55, 59). In addition, the nurses who were unable to take care of family felt guilty and unbearable, and they were still concerned about health condition of their family (12, 13, 31, 50, 56, 84). In order not to worry their family, some of them hid the fact that they worked in the front line from their family (34, 50, 53). Separation from family and minimum communication with colleagues were causes of depression (43). For nurses with children, they also experienced various psychosocial problems related to social isolation, such as fear and distress (51, 59). The anxiety of nurses who experienced social stigma intensified (73).

“*We frequently needed to deal with patients, which increased the chance of infection, but doctors spent much less time in the ward. “(N4, P6)* *(**38**)*

“*I have been most concerned about my family since this period began. Anyway, every time I come here, the environment is polluted, and there is a possibility that I will be the carrier of this disease.” (N11, P5)* *(**12**)*

“*I did not want to emotionally burden my husband during the pandemic. I did not let him know that I was working in an isolation ward... I applied for subsidy from my hospital so that I could rent an apartment...” (P8)* *(**34**)*

When working on the front line, informing good news to patients was the happiest moment for both patients and nurses (84). However, the COVID-19 patients' bad condition and announcing bad news to patients' family members could make nurses feel distress and powerless (9, 14, 23, 28, 33, 38, 40, 50, 70, 72). Sometimes, there was nothing to do but watch the patients suffering (30, 31, 35, 38, 49, 50, 66, 82). Patients with COVID-19 were separated from their families, making patients, their families, and nurses sad and anxious (74, 83). The increased number of infections and death tolls, especially the infection or death of colleagues, also made nurses feel distress and fear of death, contributing to the emotional exhaustion (9, 14, 31, 33–35, 37, 40, 42, 43, 48, 59, 60, 63, 71, 73–75, 82–84). A few nurses might have obsessive thoughts (40, 82). The negative experiences could lead to over excitement or aggressive behavior, even fatigue, pain, or insomnia (27, 42, 59, 84). More than that, some nurses experienced a sense of despair and even wanted to quit a job (16, 33, 42, 56, 59, 71, 76).

“*And at those moments, there's nothing to do…you have to stand there…be helpless (crying).“ (N4, P3)* *(**66**)*

“*During the difficult time, my colleagues had crashed, including feeling depression. I felt that I was powerless and collapsed due to high-intensity work and patients kept passing away in front of me. Many people (nurses) burst into tears after getting off work and felt that they could not keep going.” (P5)* *(**33**)*

“*My colleagues passed away, will we be the next one?” (P5)* *(**33**)*

“*Everyone is dying, who cares, this is too much work, the death rate is increasing, I wish no one would be sad, many of our colleagues died, it is hard to believe this number of deaths, the cemeteries have no place anymore. ” (N5, P1274)* *(**9**)*

“*The situation is such that many colleagues don't want to come to their shifts.” (N11, P7)* *(**42**)*

Category 4: Being positive gradually.

As time went on, nurses became relaxed and confident because of the experience of successfully managing patients with COVID-19 and correct use of PPE (16, 27, 59, 73, 78, 81). With more patients improved and recovered, nurses' psychological problems were reduced than before (33). They adapted to the work and took pride of their work again (25, 34, 50, 58, 63, 66, 74, 83). The nurses were happy and grateful to be able to help patients through their profession gradually (32, 48, 58, 66, 75), although some of them were worried and scared at the same time (75). The nurses became confident in prevention and control of the COVID-19 pandemic, and they were also calm about rescue tasks (25, 26). With multifaceted support from government, the family and the team and the respect from patients, they were happy, inspired, and grateful (13, 16, 22, 26, 27, 30, 34, 37, 47). The positive feedback encouraged nurses to work harder than before (36, 37, 48). Some nurses felt lucky that they and their family were not infected during the pandemic (62).

“*In the end, you slowly start to come to terms with the situation. This is part of our job. Now, we are more comfortable than before, believing that at least we have done our best by taking protective measures. I mean, we have embraced it a bit more. We started to see the good and bad courses of the disease. Now, we feel a little more at ease.”(P7)* *(**59**)*

“*Now, there is a lot less fear. After caring for infected patients for seven months, I believe I've gained a certain level of competency in caring for infected patients, and I can do the job when such an infectious disease outbreak occurs again.”(P14)* *(**16**)*

“*…On the one hand, I feel grateful to be working, to contribute professionally. On the other hand, I feel apprehension, frustration, impotence, and anger at living with the uncertainty of not knowing who is a suspect for the coronavirus, be it patients, co-workers, and even myself.”(P5)* *(**75**)*

“*Many people donate food and fruits to us. I appreciate it.” (N8, Pe795)* *(**22**)*

Synthesized finding 2: There were various difficulties and challenges in caring for patients with COVID-19, including fear related to providing patients with care, shortage of protective equipment and manpower, and negative attitude of family members.

Category 5: Nursing care during the COVID-19 pandemic.

For novice nurses, they had to learn how to work before their shift started (70). Due to infection of colleagues, some novice nurses suddenly became the senior nurses in their ward and provided nursing care for patients with COVID-19 (14). Once a colleague was diagnosed with COVID-19, the nurses who had close contact with the confirmed case needed to be isolated (16). The nurses who cared for patients with COVID-19 needed to be tested for COVID-19 regularly (16).

“*I went to the unit days before my shift started, to get settled in and check out how the unit works. However, it is impossible when they call you like this, at 10 a.m. to be at the hospital at around 11 a.m.” (N11, P5)* *(**70**)*

When the patients with COVID-19 were admitted to the hospitals, routine nursing practices, such as measurement of vital signs and patient education, were provided by nurses (58). During the COVID-19 pandemic, the nurses had to wear PPE while providing nursing care, and some of them spent less time in COVID-19 units to reduce the risk of infection and the frequency of patients-nurse communication (46, 58, 77, 83). It was found that many nurses provided nursing care with fear, especially when performing high-risk procedures (32, 60). The patients also needed to follow protective measures, such as wearing masks and quarantine until 14 days after discharge (58, 73).

“*The first time the patients are assigned a room, they are informed about the use of nurse call buttons at the bedside. We tell them that when they want to contact us, they should not leave the room but use the nurse call buttons, and that, when they press the button, we will take the necessary precautions before coming to their room.” (N1)* *(**58**)*

“*They cough while connecting the valve during the aspiration procedure. Therefore, we perform this procedure with fear.” (N4, P317)* *(**60**)*

The patients with COVID-19 suffered too much due to the nature of disease and isolation, and they needed support in many aspects (32, 66). The nurses not only paid attention to patients' condition but also provided constant explanation, psychological support, and all basic nursing to the patients and tried hard to meet the patients' demands and reduce the patients' negative emotions (10, 13, 16, 22–24, 32, 49–51, 59, 68, 71, 77, 79, 84). However, some patients did not cooperate with nursing care (16, 32). Violence, stigma, and no social isolation made the nursing care more difficult (75). Some nurses reported that their nursing activities and the professional role changed (38, 69). Working in isolation wards even changed the nurses' perceptions of time and space (68).

“*We need to help patients stay positive…[some patients] start crying, and we need to go into the isolation room in full gear to help them calm down…They feel scared as they don't know what's going on…stressed as [there are] a lot of unknowns…need to spend some time with them. “(N2, P5)* *(**32**)*

“*Some jobs we did should have been finished by the doctors. Some doctors even asked us to do it through phone calls.” (N9, P6)* *(**38**)*

Category 6: Negative social experience.

The great majority of nurses are supported by family, friends, patients, and community (11, 12, 16, 23, 30, 34, 36, 47, 48, 50, 51, 84). People called nurses “heroes”, although they did not feel like heroes. They were uncomfortable and thought it was weird (66, 67). A small number of nurses were not understood by family and friends (57, 64, 65). Some of them were encouraged to leave the job by their family (45, 50, 52). Numerous nurses suffered social stigma due to working in COVID-19 units (11, 12, 16, 48, 49, 51–55, 57–60, 66, 76, 78, 80). Some of them did not even want people know their work to avoid social stigma (80). When seeking for psychological support, several nurses were even stigmatized as crazy (75).

“*It's interesting that my father asks me to get away from these patients and recommends me to leave my job. He asks me if I am short in money that I have to care for these dying patients in the deathward. He says I would lose my life. For these stigmata, all my colleagues want to change their workplace and go to another ward”. (N22, P7)* *(**52**)*

“*When I left the ward, everyone was running away from me, even my relatives were staying away from me, and it was not pleasant.”(N6, P4)* *(**48**)*

“*They treat us like lepers, they think we are infecting them” (N14, P539)* *(**49**)*.

“*I can't tell others about working at this hospital because when I asked the taxi driver to take me to the hospital, he asked me if I worked there, and then he told me to get out. They don't even deliver food to the hospital. I wasn't infected with COVID-19, and I didn't do anything wrong, but I had to stay at home. Because people don't want contact with me.”(P9)* *(**16**)*

To avoid the risk of transmission, they spent much time staying at the hospital or had to be self-quarantined (11, 12, 14, 16, 29, 32, 34, 41, 42, 48–51, 53, 54, 56, 59, 60, 66, 75, 79, 80, 83, 84). It was reported that social isolation negatively affected nurses' family and social relations, education quality of children, and work quality (9, 32, 46, 49, 51, 53, 59, 80). Several nurses even experienced social deprivation (12). For overseas nurses, they were far away from family and very homesick (77). Those nurses' families were also concerned about their safety (77).

“*When I come home from work, I isolate myself at home. I don't eat with my family; I don't hug my baby” (N11, P539)* *(**49**)*

“*I cannot meet my family, we are all in the ward, it is very difficult to be away from my family, everything weighs on me, it is very hard, now think that I will come and think about my care theory, no one can think about these things in this tragedy. “(N12, P1274)* *(**9**)*

“*I've made sacrifices when it comes to my children, for example… Unfortunately, because lessons and school attendance were all online, I couldn't help my children at all, because I had to stay away. They had to do some things by themselves.” (P6)* *(**59**)*

“*Homesickness is always there. I miss my child.”(N4)* *(**77**)*

Category 7: Ethical issues in caring for patients with COVID-19.

In the initial days, the number of patients increased sharply, and PPE was inadequate. The patients' emotional needs and patient rights, such as the rights to know and the rights to personal security, were always neglected (38). Spending more time on self-protection and less time with patients could reduce the risk of infection (38, 46, 52, 77), but which could negatively affect quality of nursing care (38) and nurses' professional responsibility (52). There was a lack of compassionate care and spiritual support in providing nursing care for patients with COVID-19 (52). Due to a lack of knowledge and fear of infection, some could not manage patients on their own and had to change nursing approaches (14, 78), and some believed that nursing care was not adequate (40, 54, 56, 69, 83, 84). Furthermore, limited knowledge and skills could also decrease quality of nursing care or even lead to death of patients (52). The majority of patients with COVID-19 died without family members by their sides. End-of-life care and bereavement care should be provided for dying patients and their families (52, 67–69, 72, 74).

“*Some of the critical patients were not able to communicate, so we could not explain treatment plans to them. They could only accept what we offered.” (N2, P5)* *(**38**)*

“*Because patients have coronavirus, I can't stay with them long. Talking with a mask also makes me short of breath and, I can't communicate verbally with them as I do with other non-coronavirus patients, and that upsets me a lot.”(**46**)*

“*The patients in this ward are in dire need of spiritual care, which unfortunately is not available right now, and that is why COVID-19 patients suffer from spiritual distress”. (N24, 75)* *(**52**)*

“*Seeing these people die in total solitude struck me very much as they had absolutely no way to communicate with relatives or with the people important to them. There were only us.” (N3,P5)* *(**68**)*

Nurses reported that nursing care and treatments provided for patients with questionable benefits needed to be discussed more (72). These treatments could be painful, but which provided families with hope that the patient could survive (72). Nurses wanted more open discussions with families and all team members to achieve consistency in terms of the patients' prognosis (72).

“*... Constant ethical dilemma of keeping someone alive when there is no hope for recovery and keeping patient alive at the cost of the patient's comfort, i.e. constantly inflicting painful procedures.”(N52, P9)* *(**72**)*

Category 8: Challenges related to PPE.

There was a severe shortage of PPE and facilities, and some PPE were not qualified for the protective purpose and were of poor quality (9, 11–14, 28, 32, 34, 35, 42–45, 47, 50, 51, 56, 63, 66, 72, 76, 80, 81). Managers sometimes had to ask nurses to reuse the PPE or make do with what they had (66, 72). When hospitals had difficulties in acquiring the PPE, the standard of PPE always downgraded (32). Some nurses were treated unfairly in receiving PPE (42). To save PPE, the nurses did not dare to drink or eat for hours to avoid going to the toilet while on duty (22, 34, 40–42, 49, 60).

“*We do not have enough facilities, there are few basic facilities, this ward is not similar to an isolated ward.” (N9, P1275)* *(**9**)*

“*When you enter the ward, all you get is an apron, a pair of gloves, and a mask, and throughout the shifts, they keep saying that we are short of equipment. I agree that in these conditions we need to conserve, but lack or unavailability of equipment makes caring for coronavirus patients a challenge” (N21, P6)* *(**43**)*

“*I was angry about it [PPE re-use] and upset about it, but now that we've been doing it for months on end, it's kind of just become the norm.”(N4, P4)* *(**66**)*

“*... It's very hard to work in coveralls. I don't even drink water to avoid going to the bathroom.” (N1, P318)* *(**60**)*

Putting on and taking off PPE took nurses' a lot of time and energy, resulting in fatigue (13, 40, 58, 59, 67, 75). Wearing PPE made the nurses feel restless and only when they removed PPE did they feel relaxed (84). Wearing PPE for a long time also increased the nurses' physical burden (25, 33, 37, 46, 48, 53, 58, 59, 66, 68, 77, 84) and made them feel fatigue, discomfort (12, 16, 22, 23, 30, 33, 35, 40–42, 48, 49, 51, 59, 60, 66, 74) and resulted in complications, such as skin damage and physical injury (23, 35, 42, 48, 51, 58–60, 84). Some even experienced severe symptoms of chest pain, headache, or even dyspnea (27, 33, 35, 44, 51, 53, 59, 60, 66, 74, 84). However, there were not even enough places to bathe after the shift (75). Wearing PPE made them walk clumsily, and their protective goggles became blurred quickly. All of those affected nurses' performance (13, 16, 33, 42, 50, 58–60, 77, 79, 84) or even caused job errors (45). It was more difficult for novice nurses in performing nursing operations due to limited clinical skills (71). Wearing PPE also limited the establishment of a good relationship between patients and nurses (59, 66, 67, 84). Meanwhile, the standard procedure of donning and undressing PPE needed to learn in short time (14, 25, 79). However, there was a lack of accepted guidelines for the use of PPE at the early stage of pandemic (54, 73). Due to the surge of patients, the number of negative pressure and isolation rooms was also not enough to accommodate patients (32).

“*I sweat after wearing the protective gear for a while or when I move, such as turning patients. Then I feel clammy.” (N8, Pe794)* *(**22**)*

“*...that goggle.., put on a cap.., it's too much to bear, the day before I had a headache for a day and a half or two... and back pain, we walked in boots..., It's hard.., there is a big discomfort in the body...”(N3, P6)* *(**84**)*

“*The equipment they gave us was of poor quality. Meanwhile, in long-term use, we have problems with nutrition and rest. Most of the time, we get headaches, nausea, skin allergies, and heavy sweating at the end of the shift. It's hard, and I felt like I was dying.” (N3, P5)* *(**51**)*

“*... Because the safety goggles and the face shield are misting up, I find it very difficult to see during procedures such as starting an IV.”(N1, P318)* *(**60**)*

Category 9: Challenges related to the staff shortage and workload.

Due to the surge in the number of infections and a lack of manpower, nurses reported the nurse-patient ratio was unbalanced and workload was overwhelming (28, 30, 34, 35, 37, 42, 43, 45–48, 51, 57–59, 63, 65, 69, 71, 72, 75, 76, 84). The patients' condition might change rapidly, which increased the nurses' work pressure (42).Work pressure, the nature of work, and lacking of rest made the nurses feel exhausted and headache (12, 16, 22, 28, 31, 41, 62, 67, 74, 84). Consequently, these physical burdens could lead to a decline in work quality or nursing errors (24, 41). The nurses working in ICU experienced sleep disturbances (66). Health issues like circadian rhythm disorder or abnormal weight loss appeared (31). The nurses in COVID-19 units had to train other inexperienced nurses, which increased their workload and affected quality of nursing care (75). In intensive care unit, the shortage of staff and the increased number of patients could lead to mishaps and accidental deaths (72).

“*First, there is insufficient staff, because a lot of patients are very heavy, and they need us to care for them…”(N14, P1386)* *(**28**)*

“*There are more than 50 beds. But only two staff are on duty at night.”(N3, P4)* *(**81**)*

“*Sometimes, I felt very hungry but had no appetite, and I had lost 5 kg in the last month.” (N26, P5)* *(**31**)*

“*COVID-19 originates physical overload, so it requires more from professionals. Every day we have prone position, supine position, patients have skin injuries, it is really hard.”(N19, P4)* *(**74**)*

Deployment of nurses could alleviate the staff shortage, but it disrupted the maintenance of staff relationships (64). The new workplace, the new nursing pattern, and working with different team members could be challenging (14, 22, 24, 30, 31, 35, 53, 58, 61, 64, 71, 75, 81). It was understood that several nurses were abused by selfish behaviors of their colleagues and managers (57).

“*Anew group of nurses has been transferred in so that we can take turns off. However, their major is different from us. The cooperation is not very smooth, sometimes messy.” (N8, P4)* *(**30**)*.

“*I think communication was definitely a challenge at first, being able to have two cooks in the kitchen and understanding what role each of them played in the patient care I think was difficult.”(P3)* *(**64**)*

Category 10: Limited knowledge and skills of nurses.

According to the severity of COVID-19, many patients required mechanical ventilation with a ventilator or advanced life support. For most nurses, providing care for patients with COVID-19 was different from their previous work. All of these challenged the knowledge and skills of the nurses, especially for those who were newly graduated nurses or lacked working experience in infectious disease or intense care unit (14, 22, 25, 28, 33, 34, 38, 65, 70, 71, 74, 79, 81). For patients with comorbidities, children and conscious patients, the nurses had difficulties in providing nursing care for reasons such as patient characteristics (59). The different customs across the world should be taken into account (72). Language barriers with foreigners as well as informing breaking bad news to patients from different countries were challenging (23, 66, 84). Furthermore, several nurses worried about the possibility of forgetting their previous knowledge and skills due to only working in COVID-19 units (71, 73).

“*I don't have the experience of working in the Intensive Care Unit, and I don't know much about the use of the ventilator… Under pressure and on the verge of collapse, we all have to work in high concentrations.”(N10, P1386)* *(**28**)*

“*Because the diseases of critical patients are also very complicated, some patients, for example, have leukemia, we don't know how to do with it.” (N4, P4)* *(**81**)*

“*The majority of the population do not speak English, enough to communicate to have a conversation, but not enough with the tools we have to understand that ‘hey' you're very sick.”(N5, P4)* *(**66**)*

Category 11: Insufficient public education.

The authorities failed to control the situation by timely public education and taking measures in the initial days (32, 43, 44). The public were unaware of the severity of the pandemic and importance of preventive precautions, resulting in ignorance and noncompliance of social distancing instructions and wearing masks (12, 40, 80, 82). Meanwhile, the governments did not report the actual number of infections and death tolls, which misled the public and adversely affected implementation of precautions (44). Sometimes, there were rumors needed to deal with, and the nurses needed to provide correct information (29, 47, 51). The hygiene beliefs might be different around the world, and incorrect hygiene belief of the public could cause additional health problems (43).

“*Now we know that the virus is spread by droplets…but people [the general public] don't have any clear information on what precautions to take against this…I always see people in the bus…touching everywhere and not being that aware.” (N22, P7)* *(**32**)*

When nurses saw careless people, they thought that these people put them at a risk of infection (60), and they were angry (82). The protective measures were very important and effective (32). The public should educate themselves and follow the social distancing instructions and preventive precautions actively (46, 67). Some businessmen hoarded the PPE and raised the price, which posed a greater threat to the public health (43). The government should do more for public health protection (32).

“*...it is maddening how everyone is being careless and mindless...the people are still acting as if nothing is wrong with the pandemic and the country and they just want to go to cafes and the government has been only taking impulsive decision that is only making things worse not only for us but also for the people... no one is helping... no one” (N7, P5)* *(**82**)*

“*Working conditions are very difficult, and it'll be harder if people don't cooperate. We expect people, by staying at home, and observing personal health and social distancing, to help us and not let the treatment staff's effort be in vain.”(Pe87)* *(**46**)*

Category 12: Inefficient management of healthcare system.

There were no emergency plans for infectious diseases and no training or drills about responding to crises, resulting in a poor response (43). Inadequate supply of PPE, equipment, and high-quality services hindered the control of the pandemic (43). The coordination between auxiliary departments and clinical departments was poor (30). The contradictory information and the unsatisfactory update frequency of protocol confused the nurses (12, 47, 49, 51, 59, 61, 66, 72) and negatively impacted patients' clinical outcomes (12, 51, 59, 61). The constant changes of guidelines or protocols were not based on evidence, leading to distrust of management (72). Physicians always ignored guidelines about testing for COVID-19, which increased risk of infection (72). There was a lack of direct supervision of authorities, and the supervisors could not obtain proper feedback on the real situation (44).

“*There were never any drills for the nursing personnel of organizations managed by the Ministry of Health to prepare them for infection crises. There are only occasional mock drills for dealing with a crisis for the nurses and doctors at military hospitals. Well, we hadn't been trained and this affects our handling of this crisis as well as ability to provide effective care.” (N18, P6)* *(**43**)*

“*The departments of laboratory, radiology and pharmacy cannot follow with clinical steps. We cannot improve our efficiency.” (N9, P4)* *(**30**)*

“*The guidelines about which mask to use in which situation seems to be continually changing. With things constantly changing it makes it difficult to stay on top of things as well as you question the reasons behind some of the changes.” (N64, P5)* *(**72**)*

Synthesized finding 3: Facing difficulties and challenges, nurses could only partly cope by using mixed means to overcome those, including media, learning, gaining skills, responding together, and organizational assistance.

Category 13: Nurses' individual psychological coping styles.

To maintain mental health, several nurses took measures, such as reading, writing, music, meditation, cooking, and so on (12, 22, 23, 26, 34, 36, 37, 48, 53, 55, 59, 62, 63, 66, 74). Some increased food intake, adjusted sleep, and took vitamin supplements, and others took regular exercise to maintain physical fitness and increase the body's immunity (22, 26, 34, 48, 59, 62, 63, 66, 77, 83). However, unhealthy practices, such as increased smoking and drinking, were also used by some nurses (62, 63, 66).

“*Music helps me a lot. Uplifting music.”(N1, P5)* *(**66**)*

“*I will play my favorite online games after work, which can relieve my pressure and no longer think about my work experience.” (N1, P205)* *(**37**)*

“*I wrote in a diary every day, recording what I had done and what I had gained, and this made me feel much more comfortable.” (N12, P1139)* *(**23**)*

“*I ate a lot during the crisis. I think I gained around 10kg. I'm exhausted after work; I eat a lot so I can bulkup more energy. I took vitamins more consistently too. I do exercise as well. I limited my alcohol intake during the crisis. I didn't want my immune system to drop.”(N27, P6-7)* *(**83**)*

To avoid the impact of negative news, the nurses limited the exposure to media and paid less attention to news about COVID-19 (22, 50, 53, 67). Stop thinking or sharing the details of their experience and denying the situation was the other coping measure (30, 37, 48, 53, 59). A number of nurses took the initiative to find positive and valid information to encourage themselves (12, 26, 34, 36). Catharsis, such as crying, communicating with leaders, colleagues, family, and friends, was also useful (22, 34, 38, 53, 61, 62, 84). Some nurses dealt with stress by conceptualizing the pandemic as just another emergency and accepting the pandemic as professional responsibility and a new life style (11, 34, 50, 53, 61). The sense of professional responsibility played an important role in dealing with negative feelings and helped the nurses overcome difficulties (26, 32, 34, 36, 48, 65, 66, 73, 81, 83). The nurses' resilience and personality traits, such as challenging interest, could help them get through the pandemic (12, 50). Compassion for patients was also a kind of a psychological defense mechanism (12, 59, 66, 78).

“*… I do not watch any news in the evening, I follow them on the Internet. I muted all of the WhatsApp groups, I check them out for about 5 mins when I am available... to see if there is anything involving me... I protect myself like this …”(N5, P7)* *(**53**)*

“*I tried not to think at first. I think more in the hospital. When I come home, I go to my room and try not to have close contact with family members. I comfort myself saying that these days will pass, only some more days to go, as if it is a temporary period. At first, I was thinking a lot, so my fear, panic and anxiety were very high. Now they decreased, as I am not thinking about it.”(N7, P7)* *(**53**)*

“*One day, I felt that the working pressure was too high to bear, so I burst into tears hiding in bathroom, and I became relaxed after crying.” (N1, P8)* *(**38**)*

Category 14: Active learning through various ways.

Many nurses learned knowledge and skills about COVID-19 and protective techniques actively from the internet, social media, and team members (32, 34, 38, 55, 59, 79, 84). Selecting and redesigning the protocols about COVID-19 were beneficial for clinical practice (74). The nurses often checked and updated the modification of policies and guidelines to ensure that correct care was provided (32, 38). Focusing on patients' condition could make the nurses' overcome their fear (12, 22, 38). Although novice nurses lacked experience, they took advantages of technological skills to assist teamwork and used their creativity to solve problems and promote rescue work (79).

“*Through learning, I acquired medical nursing skill, especially for COVID-19 patients in a short time.” (N2, P7)* *(**38**)*

“*I must adjust myself because I cannot be immersed in sadness, I must focus on combating the epidemic, and take efforts to save more lives, win the battle, and help everyone to return to a normal life.” (N9, Pe795-e796)* *(**22**)*

The nurses followed precautionary measures (23, 48, 59, 75, 84), disinfected everything brought from outside (77) or even took extra measures (83) to avoid risk of infection.

Category 15: Seeking external support.

A number of nurses took the initiative to seek external support, such as medical supplies support and psychological counseling (22, 26, 71). The support from family, team members, friends, patients or social media made the nurses stay positive (34, 36, 38, 63, 65, 66, 83). Mutual support within the teams was a significant coping method (32–34, 63, 66, 67, 81, 83). Team spirit provided mutual support (14, 23, 26, 32, 34, 78). Mutual support from existing relationships could make it easier to respond to challenges in a new team, and peer support was a priority for some nurses (64). Moreover, a few nurses believed that fighting on the front line was a sign of patriotism, and this belief could help the nurses facilitate psychosocial adaptation (59). The response based on belief system was a significant strategy to deal with the negative psychological experiences (12, 48, 50, 61, 66, 67, 84).

”*We encourage each other. It doesn't feel like I'm fighting alone, I'm not afraid.”(P595)* *(**26**)*

“*[I was with] 3 of my closest friends… so just having them to be able to turn to and… bounce ideas off of or… even vent to… having some familiarity with one's colleagues also made it easier for nurses to support each other in providing patient care.”(P4)* *(**64**)*

“*I was very scared that I might pass the disease on to my family members, but I relied on God and tried to keep those thoughts away from me.” (N1, P5)* *(**48**)*

Category 16: Organization's coping strategies.

Nursing managers played an important role as coordinators and experts during the pandemic (14, 64, 84). To respond to this sudden crisis, the nursing managers adjusted the nurses' patterns of nursing care rapidly (10) and selected or employed novice nurses to form new teams regardless of previous experience (70, 79). In the hospital, temporary nurses (below 89 days) were employed (44). Furthermore, the nurses also were transferred from other departments or hospitals in local or other provinces urgently (14, 79). Managers in some hospitals established new COVID-19 wards and adapted the entire structure of original units (14, 74). General wards were converted into isolation rooms (43). Some wards even did not meet the criteria of isolation wards or lacked equipment (61). Working with new team members was challenging, so the managers often tried to cement relationship between deployed nurses and original nurses in units (64). Hospital managers created function-specific teams to deal with certain tasks to reduce the nurses' workload (64) and provided information to guide clinical practice (32, 63, 64, 67). The nursing leadership also played a crucial role in maintaining morale (63, 64). There were some financial supports, such as allowances and subsidies, provided for the nurses by the hospital (32).

“*Really, they are good. They helped us. They looked after patients as well as us. They asked what the problems are and what the shortcomings are. They arranged meetings to talk.”(N9, P10)* *(**84**)*

“*Hiring usually takes place at a very inopportune time and doesn't take into account if we are novice nurses.”(N15, P5)* *(**70**)*

“*One day, I went up to perform my normal duties and the head nurse called me. She was worried as she couldn't find nurses who could care for COVID-19patientsdue to the lack of available staff members, so she chose me to do it and I was not able to refuse at that time.”(N8, P6)* *(**79**)*

“*…the huddles at the beginning throughout it with our staff and our nurse director were very good…we could talk to each other, express [concerns],[and] it was in real time as we were going through it. You can bring stuff up, support was given.”(P5)* *(**64**)*

Category 17: Application of modern technology in caring for patients with COVID-19.

When caring for patients with COVID-19, robots were used to deliver foods, medicine, and other stuff to the patients. However, the application of robots in patient care had a negative impact on relationship between patients and nurses, resulting in the nurses' guilt (84). The nurses tried best to talk to patients in order to maintain a good nurse-patient relationship (84). Modern communication technologies, such as mobile phone, intercom system, and video camera system, were used to improve nurse-patient and patient-family communication (32, 58, 73, 74, 84). For dying patients in ICU, the application of iPads with Facetime enabled family members to say their last goodbyes, rather than leaving the patients to die alone (72). More than that, some nurses reported that patient assessment by telephone and the electronic management system were needed, which could reduce the risk of infection (16). A walkie-talkie was also used to talk to patients in order to reduce the use of PPE (32).

“*That means we do not always collide with the patients. We had a robot. He is the one who sends all the foods to the patient. We only collide directly with the patient when we take blood. That's why we have to go very rarely.” (N7, P10)* *(**84**)*

“*For elderly patients [without a phone with video calling capabilities], we give them a Tablet to use so that they can video call their family members.” (N7, P5)* *(**32**)*

“*All along the interpreter was used to communicate with this poor wife who was already stressed and overwhelmed with all that was happening. Then with IPad in hand and pressed up to the glass window of the patients' room, the family prayed, and then watched us take him off the ventilator…”(N13, P7)* *(**74**)*

Synthesized finding 4: To better respond to the COVID-19 pandemic, nurses' needs should be paid attention to. Counseling, training, information, resources, and investment are pivotal.

Category 18: The need for psychological support.

When the nurses involved in rescue work at the very beginning or suffered psychological problems, psychological support provided by psychologists and therapists for these nurses in time was necessary (16, 28, 29, 37, 45, 47, 48, 53, 59, 75, 84). It was found that emotional support should be given to the nurses (44, 60). Although some nurses reported that psychological support was available, the psychological support team did not work well (33, 63).

“*We don't know how to deal with it … I think it would be easier if there are psychologists who can provide psychological counseling …We do need some kind of support from family members or social groups because we are facing high risks.” (N2, P206)* *(**37**)*

“*…We don't know coping strategies... I feel like consulting an expert, so it would be much much better if psychosocial support were to be provided by psychologists, therapists in related fields by making appointments... We really need some sort of support, because we are under a lot of risk.”(N9, P7)* *(**53**)*

“*I think it would be nice to have counseling for nurses at the very beginning. We had nurses who did not come for the duty.”(N8, P9)* *(**84**)*

Category 19: The need for training and information.

The training project prepared the nurses well during the COVID-19 pandemic (81). Novice nurses reported that online training and on-the-job training provided by hospitals enable them to work well (79). However, there was no training or education for many nurses (28, 45, 65, 72, 81). Most nurses recognized that they had insufficient knowledge and skills to handle the pandemic of a new sudden infectious disease. The prior training of protective measures, operation of equipment, and specialized knowledge and skills of COVID-19 were needed (16, 25, 28, 32, 37, 47, 63, 69–71, 84). Regular and ongoing education and training in infectious diseases management were suggested (32). Several nurses reported that there was a gap between nursing education in school and clinical practice, and this gap should be noticed (65). The nurses should also be trained to deal with possible psychological challenges in a crisis (84). The easy and quick access to validated resources to check information was also needed (59, 70, 84). The approaches of nursing care and nursing records needed to be standardized (10), and the clear care and treatment protocols or guidelines should be provided (9, 16, 29, 72, 78).

“*I felt the training programme prepared me well for the work in the COVID-19ward.”(A6, P5)* *(**81**)*

“*…I do not even know how to protect myself; there is no related training. It's so dangerous and I feel scared when I think about this…”(N8, P1386)* *(**28**)*

“*Managers should strengthen the training of emergency rescue content in order to respond to unexpected accidents in the future.”(N2, P206)* *(**37**)*

“*If there was a clear policy about this condition that we acted based on it, it would be much better, our care is performed based on our previous experiences, I think a series of clear and scientific care and treatment policies should be provided.” (N11, P1275)* *(**9**)*

Category 20: The need for resources management.

Due to the shortage of nurses and heavy workloads, the scheduling and human resources were needed to adjust dynamically to meet the requirement of working and keep the strength of the nurses (28, 39, 47, 53, 72, 84). The communication about scheduling between managers and front-line nurses should be strengthened, and the managers should pay attention to the nurses' perspectives and needs (39). Meanwhile, it was important to increase the number of the nurses to alleviate this situation according to the infection areas' needs (9, 12, 16, 53, 71). When building new working groups and assigning nursing work to the nurses from different units, the nurses' experience, specialties, and working years should be fully considered (39). Workflow and work responsibility should be improved constantly in a new team and a new workplace (39). The physical needs of the nurses should be paid attention to and provide targeted support. It was necessary and important to meet the needs of PPE and facilities (9, 12, 29, 32, 48, 53, 57, 62, 69, 84).

“*…What we need most is an effective management team that can arrange personnel and distribute staff appropriately…”(N4, P1387)* *(**28**)*

“*Before the assignment, we were basically not asked about our work experience or anything like that.” (Participant 4, P)* *(**39**)*

“*The management of the material has been a bit chaotic, I have not felt insecure, nor that I had no material. But neither have I seen that there was enough material to provide good care.” (N16, P6)* *(**69**)*

Category 21: The needs for more investments in nursing.

A great many of nurses longed for more investment, financial support, and incentives in nursing (41, 42, 44, 46, 47, 49, 54, 57, 59, 60, 64, 71, 74, 75, 81, 84). Some of them reported that their pays were not equal to the difficulty of their work (48, 81). Some nurses hoped to improve their employee rights, such as retirement (54, 64). The nurses' need for welfare facilities, especially foods, transport, and accommodation, should be paid attention to (84).

“*I expect the authorities to support us in this situation. Well, it can be financial or promotion in the work system.”(P87)* *(**46**)*

“*You should be rewarded as a nurse for putting an extra effort into this; because it was also a risky job …it's so Florence Nightingale like. We would really like to help, you just feel that you are not rewarded for i…we would like something other than a box of chocolates for the coffee and thanks for the good treatment.”(A8, P6-7)* *(**81**)*

“*We were labelled as health heroes. We know we worked. Although labelled, we did not receive our overtime and public holiday payment even.”(N2, P9)* *(**84**)*

Category 22: The needs for the rights to be respected.

Several nurses were forced to work on the front line (49, 80, 81). They thought that their professional rights had been denied (49, 81) and hoped to be appreciated and valued by the authorities and expected the authorities to provide spiritual support for them (41, 48, 49, 54, 57, 59, 64, 84). They believed that establishment of nurses' union was crucial for protecting the nurses' rights (59). Temporary nurses under short-term employment contracts highlighted their need for job security (48). For male nurses, they hoped to be respected as a professional role (37).

“*I had to meet up (in the COVID-19ward) with 24hours warning, then they changed my employment contract again. Actually, I have no choice, I simply can't accept that.”(A4, P6)* *(**81**)*

“*We want the authorities to always pay attention to us, not when they are in urgent need. We are told that you are at the frontline of combat and you are on the battlefield against the disease. Well, this is our usual job, now it has become a little harder. So always give us importance.” (N6, P540- 541)* *(**49**)*

For decision-making regarding patient care and unit policies, the nurses believed it was a right for them, but the nurses did not involve in unit decisions (72). The nurses wished to be involved in decision-making (72). A clear explanation about the reason for decision-making was necessary for reducing the nurses' dissatisfaction (32). Communication between managers and nurses should be more available (73). Furthermore, some nurses also expected to receive some support to ensure their safety, such as the COVID-19 test regularly (69). It was also necessary to accurately reflect the problems faced by the nurses in caring for patients on the social media (59). Some nurses mentioned the need for contacts with family and friends (29). However, some nurses expressed that they just wanted to be treated and cared for as humans (62).

“*Frontline staff needs to be given more information on management decisions…if they can understand why different decisions are made, they would have fewer grievances and can be more supportive.” (N10, P6)* *(**32**)*

Category 23: The needs for improved response efficiency of healthcare system.

The system responding to the similar crisis needed to be improved (25). Timely and effective response from authorities and emergency plans for infectious diseases of healthcare system were needed (32).

Synthesized finding 5: Despite the hardships, the nurses became stronger and gained gratitude, positivity, mental peace, and confidence.

Category 24: Advancing in nursing career.

Through involving in rescue work, the professional skills, work experience, and management ability of the nurses improved (10, 14, 35, 36, 38, 46, 50, 54, 55, 57, 58, 69, 71, 73, 81, 84), and they were proud of themselves and the nursing profession (22, 30, 31, 36, 37, 54, 57, 67, 71, 73, 77, 81, 84). The scientific research ability was also enhanced (38). Some nurses learned to take care of patients with human care based on the Nightingale's thought and understood the value of nursing (41, 50, 57, 71, 76). Their empathy was enhanced (36, 81). The nurses' professional identity, professional ethics, and professional responsibility were enhanced (10, 23, 26, 28, 30, 34, 41, 50, 55, 58, 68, 81). They felt more satisfied with helping patients recover than with financial supports (57). Note mentioning is some nurses intended to improve themselves such as being specialists and pursuing master's degree (22, 23, 65, 71, 74). Unfortunately, there were a small number of nurses questioned their profession and would quit the profession when possible (57, 74).

“*I've never been so proud to be a nurse. I feel like this has been an accomplishment.”(P159)* *(**67**)*

“*I began having other objectives because there are no perspectives of professional growth for me.” (N17, P5)* *(**74**)*

“*I have become much more conscious of my competencies and value as a nurse.”(B7, P6)* *(**81**)*

“*To be honest, I once thought about quitting my job. But after this event, I feel that my professional identity has been greatly strengthened, and I have a sense of achievement.” (N7, P1139)* *(**23**)*

“*I think I have to continue to improve myself, and I suddenly have the impulse to study as a specialist nurse and a graduate student.” (N6, P1140)* *(**23**)*

Category 25: Improved relationships and social recognition.

Some nurses realized the professional solidarity between colleagues was increased, which made them support one another during the pandemic (31, 46, 54, 55, 64, 68, 77). The relationship between nurses and patients became more harmonious (23, 31, 81). The social position and perceptions of nurses improved (31, 33, 41, 46, 50, 54, 55, 59, 68, 84), and hospital managers were aware of the nurses' value (68).

“*I felt that they thanked us from the bottom of their heart. Some patients cried when they discharged from hospital. Some family members kneeled down to express their thanks when they picked the patients up.” (N9, P5)* *(**81**)*

“*One patient, when we went to facilitate his functional exercise and talk to him, was very willing to communicate with us and kept thanking us.” (N6, P1139)* *(**23**)*

“*One patient, when we went to facilitate his functional exercise and talk to him, was very willing to communicate with us and kept thanking us.” (N6, P5)* *(**31**)*

Category 26: Being more positive toward life.

After self-reflection of the nurses, they realized that their will and courage to face life enhanced, and their potential was discovered (23, 26, 57). Some recognized the importance of health and family and learned to cherish the present life and value the time and health (23, 30, 31, 36, 50, 55, 67, 77, 81). They would try more new things in the future (31). It was reported that some nurses became more patient than before (50).

“*After the epidemic, I wanted to try new things which I did not dare to do before, such as skydiving and bungee jumping.” (N5, P6)* *(**31**)*

“*Good health was the foundation of everything. I would cherish life more and pay more attention to my health than before.” (N25, P6)* *(**31**)*

“*It's nice to be alive. Everything else is unimportant.” (N1, P6)* *(**81**)*

## Discussion

In this review, a total of 70 qualitative studies were included and five synthesized findings were created to better understand the front-line nurses' experiences and needs during the COVID-19 pandemic. The front-line nurses showed a strong sense of professional responsibility and mission, and their psychological responses were dynamic and varied during the COVID-19 pandemic. The front-line nurses encountered a variety of difficulties and challenges, and they could only partly cope with. Meanwhile, they needed psychological support; training; timely, accurate information; adequate manpower, and material support et al. To better support the front-line nurses, their needs should be noticed and met. Being involved in front-line work during the COVID-19 pandemic, despite the hardships, the nurses' professional ability improved, and they gained a lot.

Not all the nurses were adversely affected, but no one was invulnerable. In our study, the front-line nurses had a lot of negative psychological experiences in the early and middle phases of pandemics, such as fear, anxiety, distress, and depression et al. According to the previous study, the prevalence of anxiety symptoms and depression among the nurses during the COVID-19 pandemic was 29 and 22%, respectively (85). A national survey in China revealed that anxiety was the most common negative psychological experience among front-line medical staff at the early stage, and the nurses reported the highest level of negative emotions (86). As time went on, the nurses showed a poorer mental health state while doctors improved (87). The psychological problems might negatively affect job performance in turn during the COVID-19 pandemic (88), and the nurses' psychological resilience might be helpful for improving job performance (89). Therefore, managers should pay attention to mental state of the front-line nurses, take steps to protect the mental health of them, and improve their psychological resilience. Firstly, regular psychological training, including self-assessment of mental state and providing psychological coping techniques, should be provided for the nurses to cope with negative emotions in the ongoing pandemic and similar crises in the future. The nurses who were psychologically prepared well for rescue work could reduce the risk of suffering mental health problems (90). As the situation progressed, the managers should focus on psychological changes and provide various forms of psychological support, including offline psychological counseling by the multidisciplinary team and online psychological support through electronic devices (91). The nurses who were younger or had fewer working years were more likely to suffer from psychological problems (92); the mental health of this vulnerable group should be extensively attended to. The nurses' psychological resilience should also be improved by training and practice. A meta-analysis suggested that resilience training could increase the nurses' resilience scores (93). The resilience training, including proper training contents and assessment tools, could be conducted for the nurses, and the feasibility and efficacy should be also considered.

According to the pieces of literature, <7% of medical staff were trained in managing patients with COVID-19; 83.8% of them lack confidence in managing suspected cases. Furthermore, <60% of medical staff received training in PPE, and only 43.2% of them knew proper hand hygiene techniques (94). In line with our findings, the nurses reported inadequate professional training. Therefore, regular and timely professional training, including knowledge, skill, and personal protective measures, and regular drills, was necessary. Timely feedback related to training and drills from the trained nurses was also important and necessary. The front-line nurses had to deal with patient deaths in the ongoing pandemic or future similar crises. Therefore, a professional training plan should include end-of-life care or bereavement care for patients and family. Simulation-based training was a practical education design aimed to improve knowledge, skill, and attitudes (95). During the COVID-19 pandemic, the simulation-based training for the nurses could improve their emergency abilities (96). These findings suggested that simulation-based training could prepare the nurses well for rescue work, which could be widely used. Nursing education in schools should provide training and drills about similar crises for nursing students to strengthen their disaster-care competencies. Calik et al. reported that a serious game, a type of technology-enhanced simulation, was beneficial for improving knowledge of infection and safe behaviors about COVID-19 among senior nursing students (97). This game was an effective teaching strategy and was free to use for purposes of research and education. School managers could employ this game for improving nursing students' knowledge and skills about COVID-19 or similar pandemics.

Health-care resource availability was associated with patients' mortality (98). However, there was a serious shortage in nurses and PPE. It was reported that workload was overwhelming due to the shortage of nurses and the surge in the number of patients with COVID-19 in our study. This was consistent with the results of Hoogendoorn et al. (99). According to our findings, these conditions could lead to reduced quality of nursing, missed nursing care, and ethical dilemma of nurses. Nymark et al. found that missed nursing care occurred more frequent among nurses working in COVID-19 wards (100). However, a comparative study reported that there was no significant difference in prevalence of missed nursing care between nurses during the COVID-19 pandemic and reference nurses (101). This might be related to the nurse-staffing level and the nurse-to-patient ratio. Therefore, effective workforce management was important. In response to the shortage of nurses, a reasonable scheduling could optimize workforce allocation and alleviate the situation of nurse shortage to some extent. When scheduling, workload, working hours, and the number of critically ill patients should be considered (102). Meanwhile, sending medical staff to the worst infection areas was a practicable solution, and a sustainable support echelon should be established. When selecting nurses, priority should be given to those with experience in working in intensive care unit or in such crisis. Some suggestions for solving the shortage of PPE are as follows. Firstly, all of the PPE should be used appropriately to prevent wastage, and the government should play an important role in reducing hoarding of PPE and making overall arrangements for existing stockpiles and donations. Meanwhile, the government could encourage medical companies to vigorously produce equipment in need and encourage other companies to shift production to making equipment, such as PPE (103). Furthermore, we found that the nurses experienced some ethical issues due to staff shortage, fear of infection, and limited knowledge and skills, et al. Regular training, psychological support, and sufficient human and material resources were also necessary to reduce ethical issues.

The results of this review showed that public education was insufficient, and the nurses had lots of negative social experiences, such as social stigma, which badly affected their work and life. Radhakrishnan et al. found that more than 50% front-line nurses experienced social stigma (104). Social stigma might be associated with insufficient public education and false information. Meanwhile, the knowledge, attitudes, and practices among the public were related to their adherence to control measure. During the COVID-19 pandemic, information about COVID-19 changed rapidly. In order to prevent the social stigma and prevent and control the pandemic, timely public education and correct information about the pandemics should be provided by authorities. Dealing with the false information timely, respecting medical staff's personality, and putting false information and beliefs aside were also effective strategies. Some technologies, such as teleconferencing and telemedicine clinics, could be used to promote the education (105). During the period of social isolation, focusing on the needs and the psychological situation of the public, and providing support could be helpful for people's adherence to control measures.

According to literature, modern technology has been used to combat the COVID-19 pandemic in recent years (106). In the current study, the nurses used modern technology to assist nursing care during the COVID-19 pandemic. These initial applications were important for future clinical practice and scientific research of modern technology. After the COVID-19 pandemic, the nurses became stronger and more positive. However, the nurses in two included studies still wanted to quit the job (57, 74). According to the Falatah' findings, the nurses' turnover intention increased after the COVID-19 pandemic (107). To stabilize the nursing team, managers should take measures to reduce the negative impact of COVID-19 pandemic or similar crises. Moreover, more investments should be provided in the nursing profession, and the nurses' rights should be respected.

## Limitations

This meta-synthesis adopted a rigorous systematic search strategy and an article review process to ensure the quality of the research. However, there were some limitations in this study. Although we searched seven databases systematically to identify related articles, it was possible to miss out the potential data from other databases. Only English-language pieces of literature were included in this study; therefore, the findings may not confidently capture the experiences and needs of the front-line nurses in non-English-speaking culture. Despite this, study settings included other cultural groups, such as in China, Japan, Turkey, Italy, Iran, Spain, South Korea, Thailand, and Brazil.

## Conclusion

This study highlighted the experiences and needs of front-line nurses during the outbreak of COVID-19. Their psychological experiences were dynamic. They also faced various challenges in rescue work and needed multifaceted support. Managers should establish psychological support services for nurses, including offline and online services for them. It is necessary to establish scientific support echelons, ensure the adequacy and availability of nurses, and provide sufficient PPE and facilities. Regular training and drills could improve emergency response capabilities of nurses.

## Implications

Managers should facilitate regular and formal training to enhance nurses' emergency response capabilities. Nurses should constantly learn to improve their knowledge, skills, and resilience. When responding to similar crises, it is necessary to establish scientific support echelons. Nurses' willingness, capacity, experience, and readiness should be considered by nurse managers. Working on the front-line was challenging; nurse managers should provide sufficient supports, including providing adequate PPE and medical equipment, and timely psychological support. Meanwhile, nurse managers should increase investment in nursing and protect nurses' rights and interests. For better work, modern technology can be properly applied.

## Data availability statement

The original contributions presented in the study are included in the article/supplementary material, further inquiries can be directed to the corresponding author/s.

## Author contributions

XY: study conception and design. SDi, SDe, and JH: data collection. SDi, SDe, ZL, and YZ: data analysis and interpretation. SDi: drafting of the article. XY and QW: critical revision of the article. All authors have read and approved the manuscript.

## Conflict of interest

The authors declare that the research was conducted in the absence of any commercial or financial relationships that could be construed as a potential conflict of interest.

## Publisher's note

All claims expressed in this article are solely those of the authors and do not necessarily represent those of their affiliated organizations, or those of the publisher, the editors and the reviewers. Any product that may be evaluated in this article, or claim that may be made by its manufacturer, is not guaranteed or endorsed by the publisher.
